# Microbial Rhodopsins: Structural–Functional Mechanisms and the Design of Optogenetic Silencing Tools—With Emphasis on *Halorubrum Archaeorhodopsins*

**DOI:** 10.3390/biology15201879

**Published:** 2026-10-18

**Authors:** Luomeng Chao, Yuxia Yang

**Affiliations:** 1 College of Animal Science and Technology, Inner Mongolia Minzu University, Tongliao 028000, China; chaoluomen@126.com; 2 College of Computer Science and Technology, Inner Mongolia Minzu University, Tongliao 028000, China

**Keywords:** microbial rhodopsin, bacteriorhodopsin, archaerhodopsin, *He*AR, *Halorubrum*, photocycle, ion pump, optogenetic silencing, retinal, protein engineering

## Abstract

Microbial rhodopsins are light-sensitive membrane proteins that use retinal as a covalent chromophore and adopt a seven-transmembrane α-helical fold. They are widely distributed across archaea, bacteria, algae, and fungi and mediate diverse functions, including light-driven ion transport, light-gated ion conduction, and photosensory signaling. Since the discovery of bacteriorhodopsin in 1971, the microbial rhodopsin family has expanded continuously, and channelrhodopsin-based optogenetics has revolutionized neuroscience by enabling millisecond-scale optical control of neuronal activity. However, compared with excitatory tools, optogenetic silencing tools have developed more slowly and remain limited by insufficient photosensitivity, poor membrane localization, ion-homeostasis perturbation, and phototoxicity. Because these properties are largely constrained by the structural and functional features of the parent microbial proteins, the discovery and in-depth characterization of natural rhodopsins remain essential for tool innovation. This narrative review focuses on ion-pumping archaeorhodopsins, with emphasis on the genus *Halorubrum* and on *He*AR, and describes how the literature was selected. We first summarize the functional classification, structural basis, photocycle, and ion-pumping mechanisms of microbial rhodopsins. We then discuss the diversity of *Halorubrum* archaeorhodopsins and the structural–functional features of *He*AR, a recently characterized archaeorhodopsin from a halophilic archaeon isolated from Ejinoor Salt Lake in Inner Mongolia, China. *He*AR absorbs at 550 nm (dark-adapted) and 560 nm (light-adapted), essentially the same maximum as AR3 (559 nm). Its Schiff-base counterion Asp-95 has a pKa of 3.5 ± 0.1, about one unit higher than BR Asp-85 (≈2.6) and AR3 Asp-95 (2.5 ± 0.2), but matched by AR4 Asp-85 (3.6); thus, this is a property that *He*AR shares with its closest relative rather than one that sets it apart. Its photocycle (≈100 ms) is prolonged—roughly ten times longer than the overall cycle of BR (11 ms), of AR4 (≈10 ms) and of the O-decay of AR3 (τ ≈ 14 ms)—while its M intermediate forms and decays faster than in BR; relative to AR3 and AR4, however, the M decay is slower (3.3 ms against ≈390 µs and 0.55/3 ms) and the overall cycle is longer. These are established in vitro properties of the heterologously expressed protein, and because a pump moves a fixed, small number of charges per photocycle, low-light performance remains a hypothesis to be tested rather than a demonstrated advantage. In this review “red-shifted” is used in one sense only: *He*AR’s 550–560 nm window lies ≈80–90 nm to the red of excitatory channelrhodopsins (≈470 nm); relative to BR it is blue-shifted by ≈18 nm, and relative to AR3 the two absorption maxima coincide. We further review the evolution of optogenetic silencing tools, from inward chloride pumps and outward proton pumps to light-gated anion channels and potassium channels, and discuss structure-guided rational design strategies, including structure prediction, molecular dynamics simulation, directed evolution, and machine learning-assisted engineering. Finally, we outline current challenges and future opportunities in phototoxicity, deep-tissue light delivery, clinical translation and the exploitation of halophilic archaeal resources, and we specify which experiments are required before *He*AR can be judged as a silencing tool. Clinically validated optogenetics is kept separate throughout from speculative applications of newly characterized archaeorhodopsins. This review aims to provide a comparative framework linking the structural–functional properties of natural rhodopsins to the rational design of next-generation optogenetic silencing tools.

## Simple Summary

Many microscopic organisms, such as salt-loving microbes, use light-sensitive proteins called rhodopsins to capture sunlight and turn it into usable energy. Scientists have borrowed these natural proteins to build tools that can switch brain cells on or off with light, a technique known as optogenetics. While tools that activate cells work well, tools that quiet cells have lagged behind, mainly because the natural proteins they come from have not been studied closely enough. This review examines how these light-driven proteins work, focusing on a new one called *He*AR, found in microbes living in a salt lake in Inner Mongolia, China. *He*AR absorbs reddish light and returns to its dark-adapted state only slowly. Both are measured properties of the purified protein; whether they make *He*AR useful for gently silencing cells over long periods is a hypothesis that still has to be tested in neurons. We also describe how researchers are using computer predictions, laboratory evolution, and artificial intelligence to design better silencing tools. We state explicitly where the evidence ends: every quantitative *He*AR value quoted here comes from heterologous expression in *Escherichia coli* and from in vitro biophysics of the purified protein. There are no electrophysiological recordings from neurons, no analysis of membrane trafficking in mammalian cells, no phototoxicity tests, no dose–response silencing curves and no head-to-head comparison with established silencing tools. *He*AR is therefore presented as a protein with interesting measured properties and testable potential, not as a demonstrated silencing tool. Finally, we keep optogenetic applications that have entered clinical testing—to date all of them retinal therapies built on animal-type opsins or engineered channelrhodopsins—clearly separate from the much longer-term prospect of using newly characterized archaeorhodopsins such as *He*AR.

## 1. Introduction

Microbial rhodopsins (type I rhodopsins) are light-sensitive membrane proteins that use retinal as a covalent chromophore and adopt a seven-transmembrane α-helical (7TM) fold. They are widely distributed in archaea, bacteria, algae, and fungi and mediate diverse biological functions, including light-driven ion transport, light-gated ion conduction, and photosensory signaling [1,2]. In 1971, Oesterhelt and Stoeckenius discovered bacteriorhodopsin (BR) in the purple membrane of extremely halophilic archaea and demonstrated that it pumps protons out of the cell in response to light [3]. In 1977, Hartmann and Oesterhelt further showed that the light-driven proton gradient established by the purple membrane is sufficient to drive ATP synthesis via ATP synthase, constituting one of the simplest photosynthetic energy-conversion systems in nature [4]. BR thus became a paradigm for biological membrane energetics and was among the first membrane proteins solved at near-atomic resolution, helping to establish the field of membrane protein structural biology [5,6].

Over the following half-century, the microbial rhodopsin family expanded rapidly. Members including the light-driven chloride pump halorhodopsin (HR) [7,8], the light-driven sodium pump (sodium-pumping rhodopsin, NaR) [9], the light-gated cation channel channelrhodopsin (ChR) [10], the natural anion channelrhodopsin (ACR) [11], the potassium-selective channelrhodopsin (kalium channelrhodopsin, KCR) [12], and the inverted-topology heliorhodopsin [13] were identified. In 2005, Boyden et al. expressed ChR2 from Chlamydomonas reinhardtii in mammalian neurons and achieved millisecond-scale optical control of neuronal excitation, marking the beginning of the optogenetics era [14,15]. Over the next decade, optogenetics was rapidly applied to neural circuit dissection, cardiac pacing, auditory research, and retinal repair [16,17,18,19].

However, compared with excitatory tools centered on ChR2 and its engineered variants, inhibitory tools have developed more slowly. In a systematic comparison in 2010, Chow et al. noted that halide and proton pumps available at the time were limited in photosensitivity, membrane localization, and silencing efficiency [20]. Wiegert et al. and Rost et al. further summarized the application bottlenecks of inhibitory tools in demanding contexts such as presynaptic terminals [21,22]. A widely recognized but still unresolved issue is that the physicochemical properties of silencing tools—pumping rate, ion selectivity, spectral window, and phototoxicity—are directly constrained by the structural–functional features of their microbial source proteins. Therefore, the discovery and in-depth characterization of new natural rhodopsins remain an essential source of tool innovation.

The salt lakes of northern China harbor abundant halophilic archaeal resources. Since 2015, the authors’ team has carried out systematic resource mining of halophilic archaea from Ejinoor Salt Lake in Inner Mongolia and has identified a series of microbial rhodopsins from multiple *Halorubrum* strains [23]. On this basis, a novel archaeorhodopsin, *He*AR, encoded by a strain highly homologous to *H*. ejinorense was recently subjected to systematic expression profiling and functional characterization. This work provided a structural–functional dataset covering absorption spectra, photocycle kinetics, counterion proton affinity, retinal isomerization, and energy-coupling efficiency [24], offering direct experimental evidence for evaluating its potential as an optogenetic tool. Because part of that evidence base comes from the authors’ own work on haloarchaea isolated from Ejinoor Salt Lake, we declare this emphasis explicitly and return to it in Section 4, where *He*AR is compared critically with AR3 and AR4 rather than presented as a privileged case.

This review focuses on ion-pumping archaeorhodopsins. It first outlines the family classification and structural basis of microbial rhodopsins, then discusses photocycle and ion-pumping mechanisms, and further compares the diversity of *Halorubrum* archaeorhodopsins and the structural–functional features of *He*AR. Subsequently, it reviews the evolution and representative applications of optogenetic silencing tools according to their silencing mechanisms, and finally discusses structure-guided rational design strategies and current challenges. The aim is to provide a comparative parameter framework for researchers working on microbial rhodopsins and optogenetic tool development. Throughout, animal (type II) opsins, non-rhodopsin photosensory proteins and device-level solutions appear only as explicitly labeled benchmarks; examples representing genuine microbial rhodopsin strategies are always identified as such.

### Literature Selection

This paper is a narrative review, not a systematic one, and the way its literature was selected should therefore be stated explicitly. Candidate papers were identified in PubMed, the Web of Science Core Collection and Google Scholar using combinations of the terms “microbial rhodopsin”, “archaeorhodopsin”, “bacteriorhodopsin”, “proteorhodopsin”, “retinal”, “photocycle”, “optogenetics”, “neural silencing”, “cryo-EM”, “XFEL”, “directed evolution” and “machine learning”, with no lower date limit so that foundational work from the 1970s onwards remained eligible. Records were kept if they were peer-reviewed primary articles or authoritative reviews reporting quantitative structural, spectroscopic, kinetic or optogenetic data on microbial rhodopsins, or quantitative comparisons between optogenetic tools. Recent major reviews and tool-comparison studies were deliberately prioritized, because they allow individual parameter values to be cross-checked, together with the primary reports on which each number rests. Work on animal-type opsins, non-rhodopsin photosensory proteins and device-level optogenetics was consulted only where a benchmark was needed, and such examples are labeled as such wherever they appear. Two limitations follow directly from this design and are stated here rather than deferred to the discussion: screening was performed at the title and abstract level, so relevant work will have been missed, and because no predefined protocol was registered, the review carries no quantitative synthesis of its own. Every reference added during revision was checked against its PubMed record, and DOIs are given in the reference list.

## 2. Microbial Rhodopsin Family: Classification, Structure, and Evolution

### 2.1. Functional Classification: Four Major Groups

According to their biological output after photoactivation, microbial rhodopsins can be broadly divided into four groups: ion pumps, ion channels, photosensors, and enzyme rhodopsins (Figure 1). Ion-pumping members are subdivided by transported ion and by the direction in which the substrate is moved. Outward pumps export their substrate and are represented by the proton pump BR [3,25,26] and the sodium pump KR2 [9,27]. Inward pumps import their substrate and are represented by the chloride pumps: halorhodopsin (HR) carries Cl^−^ into the cell [7,28], and halorhodopsin-like Cl^−^ pumps are also encoded by several marine bacteria [29]. Transport direction is not, however, fixed by the ion that is transported. Xenorhodopsins, exemplified by the rhodopsin of the deep-ocean bacterium *Parvularcula oceani*, are natural light-driven inward H^+^ pumps that keep the canonical seven-transmembrane fold; inward direction is set by the strength of the electrostatic interaction between the primary proton donor and its acceptor rather than by a different overall architecture [30]. Schizorhodopsins, discovered in Asgard archaea, form a second inward H^+^-pumping family: their crystal structure showed that the cytoplasmic halves of helices 2, 6 and 7 are shortened so that the proton acceptor lies close to the cytosol, and identified residues responsible for color tuning [31], whereas time-resolved Raman spectroscopy showed that a cis–trans thermal re-isomerization preceding Schiff-base reprotonation is common to the whole family [32]. Because an inward H^+^ pump moves positive charge into the cell, it depolarizes rather than hyperpolarizes the membrane, and this class is therefore not among the silencing mechanisms surveyed in Section 5. It is nevertheless retained here, because transport direction is a defining property of an ion pump, and because these natural inward pumps show that the canonical seven-transmembrane scaffold supports either direction—a point taken up again in Section 6.3. Ion-channel members include the early discovered cation channels ChR1/ChR2 [10,14], natural anion channelrhodopsins (ACRs) [11], potassium channels (KCRs) [12], sodium-selective ChRs [33], and the red-shifted channel ChRmine, which combines pump- and channel-like features [34]. Photosensory members are represented by SRII, which senses blue light and regulates phototactic behavior through a transmembrane transducer and a histidine kinase cascade [35,36]. Enzyme rhodopsins, discovered in recent years, contain C-terminal guanylate cyclase or histidine kinase domains [37,38]. In addition, heliorhodopsins identified in 2018 have a membrane orientation opposite to that of classical members, and their functions are still being resolved [13,39,40]. Extended discussion of these groups can be found in recent reviews [41,42,43,44].

### 2.2. Seven-Transmembrane Structure: Common Scaffold and Functional Sites

All microbial rhodopsins share a 7TM α-helical bundle formed by seven-transmembrane α-helices (A–G). *All-trans*-retinal is covalently linked to a conserved Lys on helix G via a protonated Schiff base (PSB) and is deeply buried in the hydrophobic core of the protein [5,6,42]. High-resolution structures of BR revealed the core components of ion pumping: two negatively charged counterion residues on the extracellular side (Asp85/Asp212) stabilize the positive charge of the PSB; Asp96 on the cytoplasmic side serves as the proton donor to the Schiff base in the M state; and the Glu194/Glu204 proton-release complex near the extracellular exit completes the final proton release [6,25,44]. Structures of HR and NaR showed that ion-pumping members retain the 7TM scaffold while achieving selective transport of different substrates such as Cl^−^ and Na^+^ by remodeling the ion-transport-pathway residues that line the translocation route, rather than by providing a single preformed Na+ binding site [45,46,47]. The structure of KCR revealed a potassium-selectivity mechanism independent of the canonical K^+^ channel selectivity filter [48]. Notably, rhodopsins from different species differ markedly in oligomeric assembly: BR forms two-dimensional lattice-like trimers [49], viral rhodopsins display unique structural elements [50], and ω-rhodopsins exhibit distinct functional module organization [51]; engineered mirror-image proteins further demonstrate the plasticity of the scaffold [52]. Oligomeric state has also been measured directly and without crystallization: high-speed atomic force microscopy combined with circular dichroism showed that archaeal rhodopsins are predominantly trimers whereas eubacterial rhodopsins are predominantly pentamers, the two classes giving inverted exciton couplets of the retinal chromophore [53]. Oligomeric assembly therefore co-varies with phylogeny and should be regarded as a design variable rather than a fixed property.

**Figure 1:**
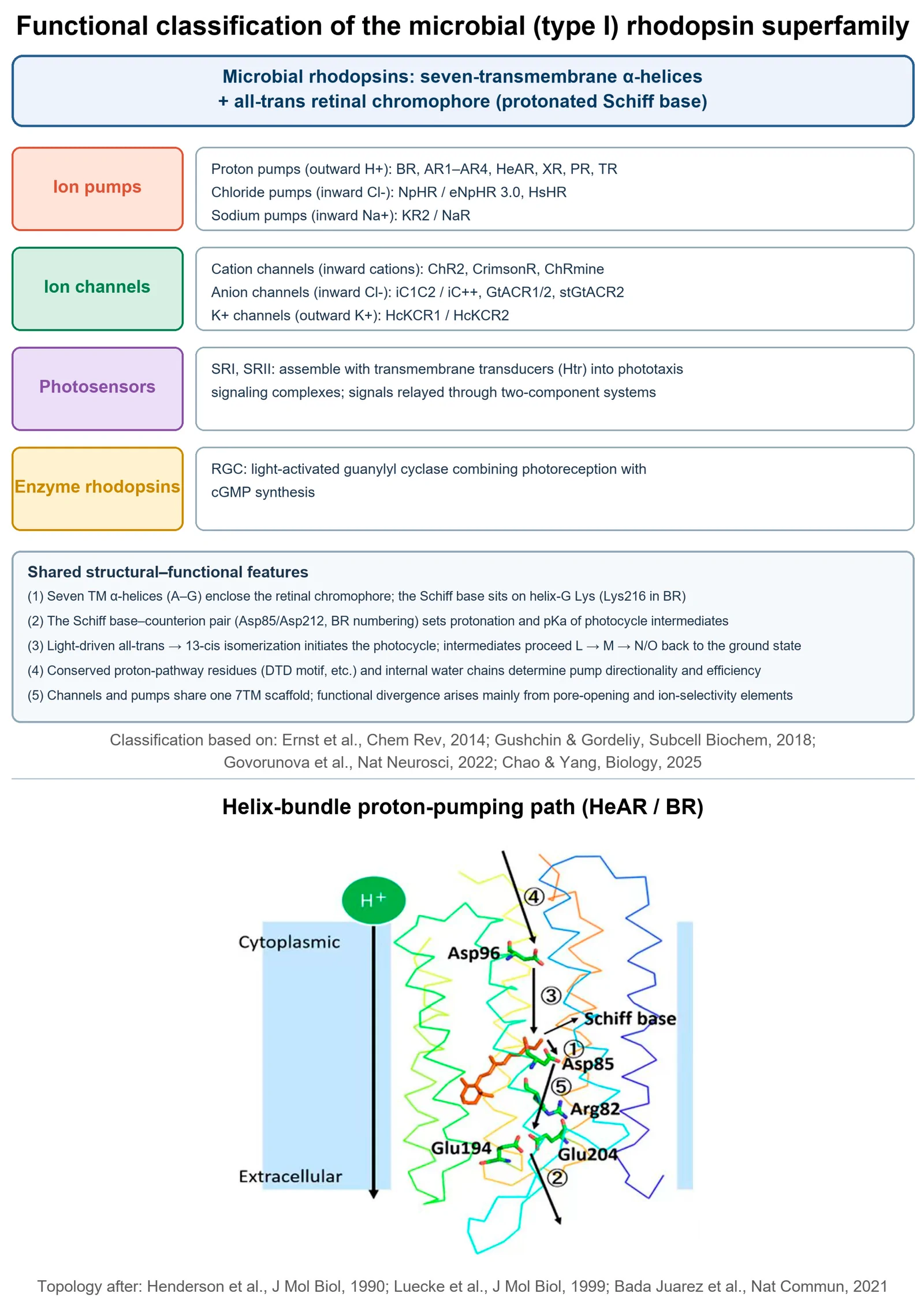
Functional classification of the microbial rhodopsin family and the seven-transmembrane structural topology. Left: the four functional groups (ion pumps, ion channels, photosensors, and enzyme rhodopsins) with representative members. Right: top view of the seven-transmembrane helix bundle of an ion-pumping rhodopsin, showing the retinal chromophore, the counterion pair (Asp85/Asp212), the cytoplasmic proton donor (Asp96), and the extracellular proton-release complex (Glu194/Glu204) [6,25,54,55]. Lower panel: the proton-transfer cascade of an outward proton pump—photoconversion of *all-trans*-retinal to *13-cis* (K), proton transfer from the protonated Schiff base to Asp85 with formation of M, reprotonation of the Schiff base from Asp96 during N, proton release at the Glu194/Glu204 proton-release complex, and re-isomerization to *all-trans* in O [6,25,44]; this panel is discussed in Section 3.3.

### 2.3. Evolutionary History and Horizontal Gene Transfer

Phylogenetic analyses indicate that microbial rhodopsins constitute an ancient protein family whose genes have undergone multiple horizontal transfers between archaea and bacteria [56,57,58]. Based on large-scale structural comparisons, Zabelskii et al. proposed that the rhodopsin superfamily can be divided into several major branches, suggesting that its functional diversity may far exceed current understanding [59]. Kojima and Sudo argued, from a photochemical perspective, that animal type II rhodopsins and microbial type I rhodopsins underwent convergent evolution [1]. Rhodopsin gene content is ecologically structured in hypersaline and marine systems. Environmental PCR surveys across a single solar saltern recovered several distinct bacteriorhodopsin-like lineages whose distribution tracked the salinity gradient rather than the taxonomy of the host [60], and a synthesis of bacterial and archaeal ion-pumping rhodopsins showed that different pump types support different light-dependent fitness strategies and display clear depth- and light-related distribution patterns at sea [61]. These field data are consistent with, but do not by themselves demonstrate, the specific proposition that pump-type and sensory-type rhodopsin genes are retained under different light regimes within haloarchaea; we therefore present that proposition as an interpretation to be tested rather than an established result. This evolutionary information provides an important lesson for tool design: natural diversity itself can be regarded as a “sequence–function mapping library” awaiting exploitation [62].

## 3. Photocycle Dynamics and Ion-Pumping Mechanisms

### 3.1. General Picture of the Photocycle

After photoexcitation, rhodopsins generally complete a photocycle in the order K→L→M→N→O→recovery. *All-trans*-retinal isomerizes to *13-cis* within subpicoseconds (K state), followed by protein backbone relaxation that drives formation of the L state. The Schiff base transfers a proton to the extracellular counterion residue, forming the deprotonated M state. Subsequently, a cytoplasmic proton donor reprotonates the Schiff base (M→N), and retinal re-isomerizes to *all-trans* in the O state, releasing the substrate ion [25,44,49]. The rate-limiting steps of pumping turnover usually lie in M/N decay and dark adaptation [63]. Time-resolved UV-Vis spectroscopy, transient infrared spectroscopy, and solid-state NMR provide complementary observation windows for resolving these intermediates [64,65,66].

### 3.2. Chromophore Isomerization and Dark/Light Adaptation

Dark-adapted rhodopsins typically contain *all-trans* and *13-cis* chromophore isomers in a characteristic ratio. Classical HPLC analysis showed that the *all-trans*:*13-cis* ratios of BR in the dark-adapted and light-adapted states are approximately 1:1 and 6:1, respectively [67]. Measurements on *He*AR showed *all-trans*:*13-cis* ratios of 2:1 and 6:1 in the dark-adapted and light-adapted states, respectively, indicating a lower enrichment of the *13-cis* isomer in the dark state than in BR [24]. More notably, the dark-adaptation half-time of *He*AR is as long as 160 min, approximately twice that of BR (80 min). Two distinct timescales must not be conflated. “Slow recovery” in what follows refers to light–dark adaptation, i.e., the re-equilibration between the *all-trans* and *13-cis* chromophore pools of the dark-adapted state (half-time 160 min; Figure 2C,D); the duration of one photocycle is a separate quantity, 100 ms for *He*AR (Figure 2A,B). Slow dark adaptation means that a larger fraction of the population stays light-adapted between illumination episodes, but because it concerns the isomeric composition of the dark-adapted pool rather than occupancy of a photocycle intermediate, it does not by itself increase per-molecule charge turnover. The proposal that it improves low-light silencing therefore needs direct measurement; Section 4.3 and Table 1 set out what has and has not been determined.

### 3.3. Proton-Transfer Cascade and Pumping Mechanism

As summarized in the lower panel of Figure 1, the structural chemistry of BR proton pumping has been elucidated in considerable detail: protonation and deprotonation of Asp85 are tightly regulated by its acid–base equilibrium [68]; the proton-release complex and imino acid residues jointly determine release timing [69,70]; and the water network in the cytoplasmic half-channel controls the energy barrier for proton uptake [71]. In recent years, room-temperature serial crystallography has directly captured water-network conformations during proton uptake [72], and computational studies have proposed long-range proton-transfer models such as the “proton cage” [73] and hydroxide-mediated transfer [74]. Femtosecond X-ray lasers have revealed the earliest structural responses of chloride pumping [75], and direct electrical measurements on proteoliposomes have enabled real-time quantification of transmembrane charge displacement [76]. These methodological advances provide a standardized toolbox for evaluating the mechanistic fidelity of newly identified pump-type rhodopsins.

### 3.4. Energy Coupling: From Proton Gradient to ATP Synthesis

The physiological significance of light-driven proton pumps lies in converting light energy into a transmembrane proton electrochemical gradient, i.e., the proton motive force, which then drives ATP synthase. This principle was demonstrated in vivo in halophilic archaea as early as 1977 [4]. Gene complementation experiments showed that the intracellular pH homeostasis and swimming behavior of purple-membrane-deficient *Halobacterium* mutants under illumination depend directly on BR expression [77]. In heterologous expression systems, *He*AR exhibits clear energy-coupling capacity (Figure 2E,F). In *Escherichia coli* BL21(DE3), photoactivation of *He*AR drives a net proton extrusion rate of approximately 0.1 ng H^+^/(mg·s). In the presence of the ATP synthase inhibitor DCCD, the net extrusion rate increases to 0.3 ng H^+^/(mg·s) because proton leakage pathways are blocked. Meanwhile, illumination for 30 s raises the intracellular ATP level to a steady state of approximately 0.3 nmol/mg protein, whereas the dark ATP hydrolysis rate is only 0.02 nmol/(mg·s) [24]. These results indicate that *He*AR is not only a subject for structural studies but may also serve as a light-energy conversion module in synthetic biology.

### 3.5. Spectral Tuning and Accessory Pigment Antennae

The absorption wavelength of microbial rhodopsins can be finely tuned by the electrostatic environment of the PSB, the water network, and hydrogen-bond strength [78,79,80]. Xanthorhodopsin (XR) has further evolved a carotenoid accessory pigment antenna that extends absorption into the blue-green region and enables efficient energy transfer to retinal [81,82]. This antenna mechanism also exists in the cyanobacterial Gloeobacter violaceus rhodopsin [83], and its expression in diatoms can significantly increase biomass production in iron-limited surface oceans [84]. Expression optimization and thermostability engineering of XR in *E. coli* have laid the foundation for industrial applications [85,86], and the energy-coupling mechanism between carotenoids and retinal has been continuously refined through theoretical and experimental studies [87,88,89,90].

## 4. Diversity of Halorubrum Archaeorhodopsins and Structural–Functional Characterization of HeAR

### 4.1. Halorubrum: A Rich Source of Rhodopsins in Salt Lake Ecosystems

*Halorubrum* is one of the most diverse genera of *halophilic archaea*, with members distributed in neutral salt lakes, alkaline salt lakes, and ancient salt mines [91,92]. Notably, in several species of this genus, rhodopsin expression is coupled with light-dependent regulation of carotenoid (bacterioruberin) synthesis, suggesting that rhodopsin-mediated photosynthetic energy metabolism may participate in the overall adaptive strategy of the cell [93,94]. Within the archaeorhodopsin (AR) family, AR1 and AR2 were first cloned from *Halorubrum chaoviator* (formerly *Halobacterium sp. aus-1*) and *Halorubrum sp.* aus-2, respectively [95,96]. Mutational analysis of AR2 first identified a stretch of polar residues at the end of helix E that is critical for light-driven proton pumping [96]. Lukashev et al. systematically characterized the pH-dependent spectral transitions of archaeorhodopsins [97]. AR4 (*Hx*BR) from *H. xinjiangense* was cloned and functionally expressed, with the recombinant purple membrane showing light-dependent photoelectric responses [98], whereas AR3 (Arch) has become the most extensively studied archaeorhodopsin because of its broad use in optogenetics [20,99].

### 4.2. Halorubrum Resources from Ejinoor Salt Lake

In 2015, the authors’ team isolated multiple halophilic archaeal strains from Ejinoor Salt Lake in Inner Mongolia and identified several microbial rhodopsin genes in its *Halorubrum* members, confirming that this salt lake is a natural enrichment site for archaeorhodopsin resources [23]. One strain showed 99.0% 16S rRNA sequence similarity to H. ejinorense, and its genome encodes three rhodopsin homologs: *He*AR, *He*HR, and *He*SRII. In contrast, *H. xinjiangense* has so far been reported to express only an AR4-like protein, suggesting that different strains within the same genus may follow distinct evolutionary trajectories in phototrophic metabolism [24].

### 4.3. Structural–Functional Features of HeAR

Sequence and phylogenetic analyses show that *He*AR has the highest homology to AR4 (95%), followed by AR1 (94%), AR3 (93%), and AR2 (86%), and only 57% identity to BR. Despite this sequence divergence, *Halorubrum* archaeorhodopsins are strictly conserved in 11 key proton-pumping residues, including the Schiff-base binding site and residues in the proton release/uptake channels, indicating strong evolutionary constraint on the functional core [24]. Site-directed mutagenesis of the same protein is consistent with that picture: replacing the consecutive polar residues Thr164 and Ser165 at the end of helix E prolonged the overall photocycle and accelerated M decay, so residues outside the retinal pocket also set the turnover rate [100]. The comparison must, however, be read with care: within this genus *He*AR differs from AR4 by only 5% of its residues and from AR3 by only 7% in the transmembrane region, so properties highlighted as characteristic of *He*AR are very likely to be shared, at least qualitatively, by its close relatives; a parameter-by-parameter assessment of what is genuinely distinctive is given in Table 1. Functionally, *He*AR recombinantly expressed in *E. coli* forms a stable 129 kDa trimer, with detergent-binding properties similar to those of BR [24]. Its absorption maximum is 550 nm in the dark-adapted state and 560 nm in the light-adapted state; the 10 nm bathochromic shift that accompanies light adaptation is a comparison within *He*AR itself, and BR shows the same 10 nm shift (568 nm dark-adapted, 578 nm light-adapted) measured in parallel [24]. Relative to BR, *He*AR is therefore hypsochromically shifted by ≈18 nm in both adaptation states (550 vs. 568 nm; 560 vs. 578 nm). AR3 absorbs at 559 nm (pH 6, *E. coli* membranes) [101], i.e., ≈9 nm to the red of dark-adapted *He*AR but within ≈1 nm of light-adapted *He*AR; because the adaptation state was not specified for the steady-state spectrum in that study, and because pH, host membrane and solubilization conditions also differ, the two proteins should be regarded as having essentially the same absorption maximum, with no red shift in *He*AR relative to AR3. The same holds against AR4: it absorbs at ≈565 nm in the light-adapted claret membrane, and the homologous protein of Halorubrum xinjiangense BD-1ᵀ expressed in *Escherichia coli* forms a purple membrane with λmax = 555 nm, so *He*AR is again slightly to the blue of its closest relative rather than to the red (Table 1). The apparent pKa of the counterion Asp-95 is 3.5 ± 0.1, significantly higher than that of BR Asp-85 (2.6) and of AR3 Asp-95 (2.5 ± 0.2; pH-dependent absorption, isomer composition and excited-state lifetimes in *E. coli*-expressed protein) [102], indicating evolutionary optimization of the electrostatic microenvironment of the retinal binding pocket [24]. AR4, however, matches it: its counterion Asp-85 titrates at pKa 3.6 (Henderson–Hasselbalch fit, n = 0.9), and every other ionizable group measured in that study is likewise higher than in BR—the proton-release complex at ≈10.4 in the ground state, ≈8.4 in M and ≈6.5 in O, and Asp-96 at ≈9.2 during the N→O transition—so a raised counterion pKa is a property that *He*AR shares with AR4 and that separates both from BR and from AR3, rather than a feature peculiar to *He*AR (Table 1). Kinetically, the complete photocycle of *He*AR is approximately 100 ms, close to those of the *Halorubrum* homologs AR1/AR2 (85 ms) and slower than that of BR (11 ms). However, its M state saturates within 60 µs (BR, approximately 0.2 ms) and decays completely within 3.3 ms (BR, approximately 11 ms), meaning that both M-state formation and decay are about three times faster than in BR, whereas the overall cycle and dark-adaptation recovery are markedly prolonged. This kinetic signature holds only against BR; against AR3 it reverses. In detergent-solubilized AR3 the M state forms within < 50 µs and decays with τ ≈ 390 µs, the O state decays with τ ≈ 14 ms, and ground-state repopulation recovers with τ ≈ 32 ms [103], so *He*AR’s M decay is roughly an order of magnitude slower than AR3’s and its overall cycle roughly three- to ten-fold longer. Because excitation wavelength and intensity, pH, temperature, detergent and the fitting model all differ between these studies, these ratios are indicative rather than exact. The corresponding measurements for AR4, made in the claret membrane at pH 7.1 and 20 °C, give an M decay with time constants of 0.55 and 3 ms, O formation and decay with 1.5 and 10 ms and an overall cycle of ≈10 ms—about four times faster than BR and about ten times faster than *He*AR; the 565 nm maximum quoted above comes from the same study [104] and the 555 nm maximum of the *Escherichia coli*-expressed *Halorubrum xinjiangense* homolog from [98]. Detergent is not a neutral variable in such a comparison: solubilizing AR4 in Triton X-100 lowers the pKa of the proton-release complex below 7.0 and reverses the order of proton release and uptake at neutral pH [105], so the physical form in which the protein is prepared can change the very property being measured. AR4 can nevertheless be produced recombinantly in *Halobacterium salinarum* L33 at yields of up to 20 mg L^−1^ with its trimeric packing and its proton-pumping kinetics retained [106]. We deliberately avoid the term bistable here. Bistability in optogenetics denotes photo-interconversion between two thermally stable states that persists in the dark, demonstrated by direct bidirectional photoswitching; *He*AR has not been shown to behave in this way. What has been measured is simply fast intermediate kinetics combined with a slow overall cycle in detergent-solubilized protein, which is a different phenomenon. Because these pumps transfer one proton per photocycle, the longer cycle lowers maximal per-molecule turnover relative to BR, so any low-light advantage must be measured rather than inferred (Table 1). Overall, of the three properties originally highlighted, only the prolonged overall photocycle sets *He*AR apart from all three comparators: ≈100 ms against 11 ms for BR, ≈10 ms for AR4 [104] and an O-decay of τ ≈ 14 ms for AR3. The raised counterion pKa is shared with AR4 (3.5 ± 0.1 against 3.6 [104]) and separates both proteins from BR (2.6) and AR3 (2.5 ± 0.2), and the “red-shifted absorption” holds only against excitatory channelrhodopsins (≈470 nm): *He*AR is ≈18 nm blue-shifted relative to BR, has essentially the same maximum as AR3, and is ≈5–15 nm to the blue of AR4 (Table 1).

### 4.4. Tool Development from Archaeorhodopsins: Lessons from AR3

AR3 is currently the most widely used archaeorhodopsin tool. Structural studies have revealed that its sensitization process is closely related to disorder of the internal water network [99]. Red-shifted and near-infrared analogs based on AR3 have extended its spectral window [107]. Its excellent fluorescence properties make it an attractive template for engineered fluorescent reporters [101], and derived voltage indicators have enabled optoelectronic imaging in tissue [108,109]. Its light-driven proton extrusion has also been used to silence synaptic transmission by altering extracellular pH [110] and even to trigger tumor cell apoptosis [111]. The success of AR3 suggests that “atypical” biophysical properties of source proteins—such as fluorescence, pH effects, and slow recovery—are often precisely the source of their value as tools. Table 2 summarizes the basic properties of representative *Halorubrum* archaeorhodopsins. Table 1 then compares *He*AR quantitatively with BR, AR3 and AR4, distinguishing parameters that have been measured from those that have not been reported in the literature and from those that were not determined in this work.

**Table 1:** A quantitative comparison of *He*AR with bacteriorhodopsin and with the archaeorhodopsins AR3 and AR4, stating explicitly what each parameter does and does not establish.

Parameter	*He*AR	BR	AR3/AR4	Source and Comparability
Source organism	*Halorubrum* sp. Ejinoor	*Halobacterium salinarum*	*Halorubrum sodomense* (AR3); *Halorubrum xinjiangense* (AR4)	[24] and references therein
Residue identity relative to *He*AR	—	57%	AR3 93% (transmembrane region); AR4 95%; AR1 94%; AR2 86%	[24]; single alignment method
Absorption maximum	550 nm (dark-adapted)/560 nm (light-adapted)	568 nm (dark-adapted)/578 nm (light-adapted); ≈18 nm to the red of *He*AR, measured in parallel	AR3: 559 nm (pH 6, *E. coli* membranes; adaptation state not specified) [101]. AR4: ≈565 nm (light-adapted claret membrane, pH 6.8, 75 mM K_2_SO_4_, 20 °C) [104]; 555 nm for the *Halorubrum xinjiangense* BD-1ᵀ homolog expressed in *E. coli* [98]	[24,101]; *He*AR and BR measured in parallel; AR3 measured at pH 6 in *E. coli* membranes, conditions not comparable
Overall photocycle duration	100 ms	11 ms	AR3: O-state decay τ ≈ 14 ms; ground-state repopulation τ ≈ 32 ms (two-exponential fit, 3.8 and 54 ms); a single total-cycle value was not reported [103]. AR4: ≈10 ms (O-formation 1.5 ms, O-decay 10 ms; ground-state recovery 0.6, 3 and 10 ms; pH 7.1, 75 mM K_2_SO_4_, 20 °C) [104]	[24,103]; 532 nm nanosecond excitation versus flash photolysis in [24]
M-state decay	3.3 ms	11 ms (M decays together with the O state; not an independent value)	AR3: τ ≈ 390 µs [103]. AR4: τ = 0.55 and 3 ms (BR, 3 and 10 ms) [104]	[24,103]
M-state formation	60 μs	0.2 ms	AR3: < 50 µs [103]. AR4: not reported separately; the M→N transition is ≈4× faster than in BR [104]	[24,103]
Dark-adaptation half-time	160 min	80 min	not reported for AR3; for AR4 only the qualitative statement that bacterioruberin modulates the dark-adapted retinal equilibrium [112]	[24]; searched for, no dark-adaptation half-time found for AR3 or AR4
*all-trans*:*13-cis* ratio, dark-adapted	2:1	1:1	AR3: 43:57 (HPLC, n = 3, pH 6) [101]. AR4: not reported	[24,101]; HPLC in both studies
*all-trans:13-cis* ratio, light-adapted	6:1	6:1	AR3: 95:5 [101]. AR4: not reported	[24,101]; HPLC in both studies
Schiff-base counterion pKa	Asp-95, 3.5 ± 0.1	Asp-85, ca. 2.6	AR3: Asp-95, 2.5 ± 0.2 (Asp-222, 1.5 ± 0.2) [102]. AR4: Asp-85, 3.6 (Henderson–Hasselbalch fit, n = 0.9); proton-release complex ≈ 10.4 (ground state), ≈8.4 (M), ≈6.5 (O); Asp-96 ≈9.2 (N→O)—all higher than in BR [104]	[24,102]; pH titration of *E. coli*-expressed protein in both studies
Order of proton release and uptake at neutral pH	not determined	release precedes uptake (sub-millisecond release)	AR4: uptake precedes release; the normal order is restored above pH ≈ 8.6, and is also restored by solubilization in Triton X-100 [104,105]. AR3: not reported	[104,105]; pH-dependent dye and transient absorption in claret-membrane suspension; *He*AR not measured
Oligomeric state in detergent	trimer, ca. 129 kDa	trimer	AR3: trimer in the native membrane, forming a hexagonal lattice as in BR (atomic force microscopy of H. sodomense claret membrane); the detergent-free three-dimensional crystal is monomeric [99]; not measured in detergent. AR4: trimer stabilized by bacterioruberin (qualitative, solid-state NMR) [112] Recombinant AR4 retains the same trimeric packing (2D solid-state NMR, DLS, FTIR) [106].	[24,112]; size-exclusion chromatography for *He*AR only
Structural coverage	predicted model; TM-score > 0.92; Cα-RMSD 1.3 Å against AR3 (PDB 6GUX)	crystal structures available (e.g., PDB 1C3W)	experimental structures available for AR3	[24]; *He*AR values compare a model with a structure
MD: chromophore hydrogen-bond persistence	80%	40%	not determined in this work	[24]; identical MD protocol applied to *He*AR and BR only
MD: protein RMSD	2.0 ± 0.2 Å	2.3 ± 0.3 Å	not determined in this work	[24]; identical MD protocol applied to *He*AR and BR only
Retinal docking free energy	−12.27 kcal/mol	computed under the same protocol	not determined in this work	[24]; the covalent docking scheme used did not reproduce the Schiff-base linkage, a limitation stated in [24]; applied to *He*AR and BR only
Isomerization quantum yield Φiso	not determined	0.64 ± 0.04	AR3: 0.54 ± 0.03 [101]. AR4: not reported	[101]; pH 6, *E. coli* membranes; not determined for *He*AR
Silencing current in neurons	not tested	—	established for AR3-derived tools	see Section 5.3
Membrane trafficking in mammalian cells	not tested	—	extensively characterized for AR3-derived tools	see Section 5.3
Phototoxicity assessment	not tested	—	partially addressed for AR3-derived tools	see Section 7.1

Note: *He*AR values are those reported for protein heterologously expressed in *Escherichia coli* and purified in detergent [24]; BR comparator values were measured in parallel within the same study, so rows marked with [24] are internally comparable. Three labels are used in the AR3/AR4 column—“not reported”, searched for and no measurement found under any conditions—and “not determined in this work”, available for *He*AR and BR because the present authors measured or computed it and did not repeat the procedure. Photocycle rates, pKa values and isomer ratios depend on excitation conditions, pH, temperature, detergent and fitting model, and AR3 and AR4 were measured under conditions different from those of the *He*AR column; those conditions are given row by row in the last column, and the values are not numerically commensurate with the *He*AR column. In row 10 the AR4 values were obtained by pH titration except the ground-state value of ≈10.4 for the proton-release complex, an estimate from the pH-dependence of M-rise [104]. The AR3 dark-adaptation half-time and oligomeric state in detergent were searched for and not found; the QuasAr1 variant, which carries five substitutions including D95H, and a survey in which AR3 appears only in the phylogeny do not report wild-type values. The last three rows list the experiments still missing for *He*AR. *He*AR, *Halorubrum Ejinoor* archaeorhodopsin; BR, bacteriorhodopsin; AR, archaeorhodopsin; Φiso, isomerisation quantum yield. The Schiff-base counterion is Asp-95 in the numbering used for *He*AR and AR3 and Asp-85 in that used for BR and AR4; both refer to the same residue.

**Table 2:** Representative *Halorubrum* archaeorhodopsins and their basic properties.

Rhodopsin	Source Strain	Functional Type	Representative Features	Ref.
AR1	*Halorubrum* chaoviator (formerly Halobacterium sp. aus-1)	Proton pump	First archaeorhodopsin to be gene-cloned	[54,93]
AR2	*Halorubrum* sp. aus-2	Proton pump	Mutational analysis identified a stretch of polar residues at the end of helix E critical for light-driven proton pumping	[94]
AR3	*Halorubrum* sodomense	Proton pump	Main optogenetic silencing tool Arch; pH-dependent spectral transitions; internal water-network disorder associated with sensitization	[20,95,97]
AR4 (*Hx*BR)	*Halorubrum* xinjiangense	Proton pump	Light-driven proton-pump function validated by heterologous expression; highest sequence homology to *He*AR (95%)	[96]
*He*AR	*Halorubrum* sp. Ejinoor	Proton pump	λmax 550/560 nm; photocycle 100 ms; M state saturates at 60 µs and decays in 3.3 ms; Asp-95 pKa 3.5; dark-adaptation half-time 160 min; light-driven ATP synthesis	[24]
*He*HR/*He*SRII	*Halorubrum* sp. Ejinoor	Chloride pump/photosensor	Two additional rhodopsin homologs co-encoded with *He*AR in the same genome	[23]

Note: Functional types and features are cited from the corresponding references; *He*AR-related data are from Ref. [24].

## 5. Evolution of Optogenetic Silencing Tools

### 5.1. Functional Requirements for Silencing Tools

Optogenetic silencing requires clamping the membrane potential below threshold with millisecond temporal precision without damaging the cell. Systematic reviews have pointed out that an ideal silencing tool should simultaneously meet several requirements: high unitary conductance, low light-intensity demand, ion selectivity, and good membrane localization [19,21]. In regions highly sensitive to ion homeostasis, such as axon terminals, the efficacy and side effects of existing tools remain particularly problematic [22].

### 5.2. Light-Driven Inward Halide Pumps

The first genetically encoded optical silencing tool was HR from Natronomonas pharaonis (NpHR), which pumps Cl^−^ into the cell and causes hyperpolarization [113]. The 1.8 Å crystal structure of *Np*HR clarified the structural basis of chloride pumping [45,46], but this tool is limited by poor membrane localization and dependence on intracellular Cl^−^ homeostasis [20]; its transport kinetics are also strictly constrained by the chloride gradient [114]. In 2010, Chow et al. developed eNpHR3.0 by adding a membrane trafficking signal sequence, greatly improving membrane localization and silencing efficiency [20].

### 5.3. Light-Driven Outward Proton Pumps

During the same period, AR3 (Arch) from *H. sodomense* and ArchT from another halophilic archaeal strain were introduced into the silencing toolbox as outward proton pumps [20,115]. Proton pumps have the advantage of being driven by a single gene and requiring low light intensity; however, they cause extracellular pH shifts and consequent confounding effects on synaptic transmission [110]. The need for red-shifted and deep-tissue light delivery has also continued to drive spectral engineering [116].

### 5.4. Light-Gated Anion Channels

Guided by ChR crystal structures, Berndt et al. converted a cation channel into a chloride-selective channel (iC1C2) by site-directed mutagenesis [117]. Klapoetke et al. further developed the high-conductance variant iC++, which eliminates residual proton currents [118]. Meanwhile, Govorunova et al. discovered natural anion channels GtACR1/2 in cryptophytes, which exhibit excellent Cl^−^ selectivity without engineering [11]. Structure-guided channel engineering has since become a paradigm [119,120,121]. Mechanistic studies of anion channels showed that their proton-transfer pathway determines rectification properties [122]. The pump-like channel ChRmine is cited here only as a structural comparator, not as an inhibitory tool: it conducts cations and is used for excitation, but by sequence and structure it occupies an intermediate position between pumps and channels and therefore delimits what mutation within the shared seven-transmembrane bundle can achieve [34].

### 5.5. Potassium-Selective Channels, Metabotropic Strategies, and Enzyme-Coupled Rhodopsins

K^+^ is a central ion in neuronal electrophysiology, but natural light-gated K^+^ channels remained elusive for a long time. In 2022, Govorunova et al. discovered KCRs (HcKCR1/2) in the stramenopile microorganism Hyphochytrium catenoides, a relative of filamentous fungi. These channels achieve a K^+^ selectivity of P(K)/P(Na) ≈ 23 through an independently evolved mechanism and mediate strong hyperpolarization within 1 ms after illumination [12]. Subsequent cryo-EM structures revealed their unique ion selectivity filter [48], providing a structural basis for engineering [123]. Earlier, strategies based on fusing natural K^+^ channels with light-controlled subunits had already demonstrated the feasibility of K^+^ conductance-based silencing [124]. Sodium-selective ChRs are also being developed [33]. Three further tools are listed for completeness, with their molecular class stated explicitly because none of them is a microbial rhodopsin ion transporter. Two are metabotropic and rely on animal-type GPCR cascades rather than on rhodopsin-mediated transmembrane charge transfer: the bistable tool optoGPCR enables multiplexed circuit silencing [125]—it is “bistable” in the strict sense, because interconversion between two thermally stable photostates was demonstrated directly, a term we do not extend to *He*AR—and eOPN3, an opsin of insect origin that, when targeted to presynaptic terminals, suppresses transmitter release through the G_i/o_ pathway rather than by transporting protons across the membrane [126]. The third is a genuine microbial rhodopsin strategy and is therefore the most relevant comparator for this review: RoCK couples a light-activated rhodopsin-guanylyl cyclase to an engineered cGMP-gated K^+^ channel, so that illumination opens a potassium conductance through an enzymatic second messenger instead of through direct ion pumping [127]. This illustrates that proton-pumping archaeorhodopsins are not the only microbial rhodopsin route to silencing. Finally, the two-color bidirectional tool BiPOLES enables precise combined excitation and inhibition control [128].

### 5.6. Application Landscape

Silencing tools have broadly empowered neuroscience and translational research. In epilepsy, optogenetic stimulation can establish an epilepsy-resistant state [129] and precisely control epileptiform activity [130,131,132,133]. In cardiology, optogenetic vagus nerve stimulation improves heart failure [134,135], and cardiac optical pacing and arrhythmia studies continue to advance [136,137,138]. In hearing research, optical stimulation of spiral ganglion neurons has paved the way for optical cochlear implants [139,140]. In pain research, sustained and reversible pain suppression has been achieved [141,142]. In sleep–wake regulation, orexinergic, REM-related, and dopaminergic circuit mechanisms have been dissected [143,144,145,146]. The most translationally promising area is vision restoration: from early proof-of-concept studies [147,148,149] to high-sensitivity visual restoration by ectopic expression of human opsins in human retina [150], and further to synthetic opsin therapy and MCO-010 therapy showing safety and visual improvement in patients with advanced retinal degeneration [151,152,153], optogenetics is accelerating toward clinical application [154,155,156,157]. Table 3 and Figure 3 systematically compare the landscape of silencing tools, including Jaws, a red-shifted cruxhalorhodopsin that noninvasively mediates transcranial inhibition of neurons deep in the brains of awake mice [158].

## 6. Structure-Guided Rational Design Strategies

### 6.1. Structure Prediction and Comparative Structural Biology

AlphaFold has advanced single-chain structure prediction to near-experimental accuracy, significantly lowering the modeling barrier for new rhodopsins, including *He*AR [159]. Visualization platforms such as ChimeraX support large-scale structural comparison [160]. High-resolution structure series represented by KR2 have revealed the molecular mechanism of sodium pumping [27,47]. Comparative studies of NaR have systematically resolved red-shifted mutations [161], the DTG motif for novel Li^+^/Na^+^ dual-substrate pumping [162], direct detection of substrate uptake and release [163], and solid-state NMR conformational analysis [164], and have revealed the functional diversity of the NaR family [165] and the applicability of nanodisc-reconstituted electrophysiology [166]. Comparable information exists for the other major functional classes and is not confined to NaR: near-atomic models of BR were among the first membrane protein structures solved [5,6]; high-resolution structures of the inward chloride pump *Np*HR [45,46] show how the shared bundle is adapted to an anionic substrate, while heliorhodopsin structures revealed a scaffold compatible with inverted membrane topology and an unexpected retinal orientation [13]; the potassium-selective channelrhodopsins revealed a selectivity filter unrelated to that of canonical K^+^ channels [48]; and structures of the pump-like cation channel ChRmine [34] show how a pump-like active site is converted into a passive conduction pathway. Cryo-electron microscopy has become central rather than exceptional in this area: the 2.0 Å ChRmine structure [34] was used directly to design variants with altered spectral and kinetic properties, establishing structure-guided engineering of a microbial opsin rather than merely its structure determination; cryo-EM of ChRmine reconstituted in lipid nanodiscs then resolved the retinal-bound and apo states and yielded variants with ten-fold slower and two-fold faster closing kinetics [167], and cryo-EM of GtCCR4 linked an outwardly bent transmembrane helix to its unusually high light sensitivity [168]. Because the stated aim is tool design, these classes are compared by what each reveals for engineering rather than ranked by the number of structures available.

### 6.2. Computational Simulation and Dynamic Structural Biology

Molecular dynamics simulation is a core tool linking static structures to function. The continuous development of high-performance simulation frameworks such as GROMACS has made microsecond-scale simulation of complete photocycles possible [169,170]. Molecular docking supports virtual screening of chromophore analogs [171]. Structural methods are more accurately classified by X-ray source than by method name: synchrotron-based room-temperature serial crystallography and XFEL-based serial femtosecond crystallography (SFX) [75,172], together with computational studies of pathological mutations in animal-type rhodopsins [173], have enriched the analytical dimensions of dynamic structural biology. Examples taken from animal rhodopsins are retained only where the insight transfers: the extracellular proton-release domain of BR and helix rearrangements in GPCRs pose the same mechanistic question of how a seven-transmembrane bundle couples chromophore isomerization to an asymmetric output, and the transferable lesson is methodological—that retinal-pocket electrostatics can be predicted and then tested by mutagenesis—rather than one of residue identity, because the two families share no detectable sequence homology. The same logic is illustrated by atomistically designed variants of microbial opsins whose absorption was blue-shifted by up to about 100 nm and then confirmed by spectroscopy, X-ray crystallography and electrophysiology [174], a case in which computation predicted a property and experiment verified it.

### 6.3. Directed Evolution and Semi-Rational Engineering

Directed evolution traverses the protein fitness landscape through iterative mutation and screening [175] and has yielded far-red fluorescent variants [176,177,178] and non-natural conformations such as soluble BR in the rhodopsin field [179]. Ion specificity can be switched by very few mutations: replacing the Schiff-base counterion Asp85 of BR with threonine converted this proton pump into a light-driven chloride pump, an early demonstration that a single residue sets ionic specificity [180]. The milestone case of functional inversion—converting a bacterial sensory rhodopsin into an inward proton pump [181]—demonstrates the high plasticity of the 7TM scaffold at the mechanistic level, and together with the naturally occurring inward H^+^ pumps described in Section 2.1 [30,31] it indicates that transport direction is itself an engineerable property.

### 6.4. Machine Learning-Assisted Design

Data-driven methods are reshaping the paradigm of rhodopsin engineering. Bedbrook et al. used machine learning to guide ChR variant design and achieved efficient optical control at low light intensity [182]. Toolchains including big-data-based color-tuning rules and absorption-wavelength prediction models [183,184], genotype–phenotype mapping databases [62,185], and ion-pump classifiers [186] have enabled an initial inverse-design workflow in which desired spectra and functions are input and candidate sequences are output. Machine learning has also been used to optimize non-Kasha photochemical kinetics [187].

### 6.5. Functional Engineering from Structure to Application

Structure–function information has also enabled diverse application-oriented engineering, including BR–titanium dioxide hybrid systems for photocatalytic hydrogen production [188], unidirectional electron injection in biogenic p–n junctions [189] and industrial-scale high-yield BR fermentation [190]. Three qualifications are necessary. First, the BR–lipid studies did not set out to engineer a rhodopsin: they characterized how native archaeal lipids and detergents modulate trimer dynamics and proton release/uptake [191,192]; we cite them only for the design insight they afford, which is our interpretation of their results and not their stated aim. Second, intracellular rhodopsin-based control of metabolism and cell signaling is indeed a microbial rhodopsin technology [193,194,195]; that list deliberately excludes Ref. [196], which describes optogenetic control through LOV-domain photoswitches and therefore belongs to a different protein family (see Section 5.5). Third, molecular engineering is separated from device engineering: wireless near-infrared-powered implantable devices are device-level solutions that extend the toolchain without altering the rhodopsin itself and are treated in Section 7.2 [197].

### 6.6. Implications of HeAR for Silencing Tool Design

Based on the available data and progress in the field, *He*AR offers at least three implications for silencing tool design. First, the coupling between M-state kinetics and silencing efficacy merits reassessment. The M-state formation (60 µs) and decay (3.3 ms) of *He*AR are both about three times faster than those of BR, whereas its overall photocycle (100 ms) and dark adaptation (160 min) are markedly prolonged. Whether this combination actually enables sustained steady-state pumping at low light doses is not established: a pump moves a fixed, small number of charges per photocycle, and the prolonged overall cycle lowers the maximal per-molecule turnover, so low-light performance has to be measured rather than inferred from the kinetic signature (see Section 4.3 and Table 1) [24]. Second, the high proton affinity of Asp-95 (pKa 3.5) implies that the counterion of *He*AR remains deprotonated within the physiological pH range, which may help maintain spectral and kinetic stability under extracellular environmental fluctuations [24]. Third, the 550–560 nm activation window of *He*AR lies ≈80–90 nm to the red of mainstream excitatory tools (around 470 nm), providing spectral compatibility for multichannel applications [24]. This separation, and not any red shift relative to BR (568/578 nm) or AR3 (559 nm [101]), is the spectral property on which that compatibility rests: relative to BR, *He*AR is blue-shifted by ≈18 nm, and relative to AR3 the two absorption maxima coincide to within a few nanometers. These properties make *He*AR another archaeorhodopsin scaffold with engineering potential after the Arch family. The route is not without precedent within the genus: AR4, which is 95% identical to *He*AR, has been produced recombinantly in Halobacterium salinarum L33 at yields of up to 20 mg L^−1^ with its trimeric packing and proton-pumping kinetics retained, and characterized in situ by two-dimensional solid-state NMR [106], so the expression, stabilization and spectroscopic characterization steps that a *He*AR-based tool would require have already been demonstrated for a protein of the same genus. Future work should focus on enhancing unitary conductance and optimizing membrane targeting based on its structure.

## 7. Challenges and Perspectives

### 7.1. Phototoxicity, Chromophore Homeostasis, and Tool Stability

Illumination inevitably introduces risks of oxidative stress and thermal damage. Intelligent illumination strategies such as closed-loop optical control can significantly reduce cumulative light dose [198]. Chromophore homeostasis is another often underestimated constraint: ChR2 degrades faster under retinal-deficient conditions [199]; intracellular accumulation of *all-trans*-retinal is cytotoxic and requires pharmacological protection [200]; and opsin folding efficiency can be improved by molecular chaperone strategies [201]. In addition, the expression and functional maintenance of tool proteins under physiological states such as aging require systematic evaluation [202].

### 7.2. Light Delivery and Deep-Tissue Applications

Strong scattering and absorption of visible light by mammalian tissue limit the in vivo application of optogenetic tools. Strategies to address this include red-shifted and far-red tools [116,203], upconversion nanoparticle-mediated near-infrared activation [204,205,206], bioluminescence-driven optogenetics without external light sources [207], two-photon excitation [208], and integrated optrode–electrode recording devices [209]. Engineering innovations are also advancing rapidly in photosensitive tumor therapy [210], smooth muscle optical control [211], all-optical readout [212], voltage indicator optimization [213,214], and simplified mRNA delivery [215]. Systematic translational assessment can be found in Ref. [216]. These are device-level rather than molecular-level solutions, and we classify them as such: in photovoltaic-powered implants the near-infrared light serves only as a power source for a local blue-light emitter that in turn activates ChR2, so the rhodopsin is not itself excited by near-infrared light [197].

### 7.3. Opportunities and Barriers in Clinical Translation

Clinical validation of optogenetics has entered a critical window. MCO-010 therapy for Stargardt disease has completed the first open-label safety and efficacy study in the United States [152]; synthetic opsin therapy has restored functional vision in patients with advanced retinal degeneration [151]; and efficient and sustained sensory and cardiac optical control elements have been validated in small-animal systems [137]. However, dose control of gene vectors, long-term immune and safety evaluation, and establishment of regulatory pathways remain practical barriers to translation [154,216]. Two features of this clinical record must be kept in view. Every construct tested in humans so far is an animal-type opsin or an engineered channelrhodopsin; no archaeorhodopsin has entered clinical testing. None of the newly characterized archaeorhodopsins discussed here, *He*AR included, has been validated even in neurons. Any prospective *He*AR application therefore inherits all the generic barriers above plus several additional layers of untested assumption, and cannot reasonably be placed on the same timeline as the retinal therapies described here. Optogenetic brain–computer interfaces and expanded applications in plant and Drosophila model systems offer broader possibilities [217,218,219].

### 7.4. Halophilic Archaeal Resources: An Underexploited Tool Reservoir

Although metagenomics continues to produce new rhodopsin sequences [220], including contributions from Antarctic phototrophic ecosystems [221], flotillin-related rhodopsins carrying pigment-free binding sites [222], and the ω-family [51], functional characterization based on culturable halophilic archaea remains irreplaceable. Northern China contains numerous salt lakes that are natural enrichment sites for microbial rhodopsins. The authors’ team’s work on Ejinoor Salt Lake shows that in-depth characterization of a single strain can yield a complete structural–functional parameter set with tool value [23,24]. Combining Raman spectroscopy-based single-cell typing [223], culturomics, and functional genomics should enable large-scale discovery of new pump-type rhodopsins with unique spectral windows, salt–alkali stability, and high pumping efficiency, providing a continuous source of molecular templates for rational silencing tool design.

## 8. Conclusions

Microbial rhodopsin research has spanned more than half a century: from the phenomenon of light-energy conversion in the purple membrane, through the chemical elucidation of the atomic structure and ion-pumping mechanism of BR, to the explosive expansion of the optogenetic toolbox. This trajectory exemplifies the classic path from basic research to technological translation. At present, the iterative frontier of silencing tools is advancing along three directions: diversification of ion selectivity, customization of spectral windows, and precision of kinetics. Each generation of tools has been enabled by a deeper understanding of the structure–function relationships of natural rhodopsins. The continued discovery and in-depth characterization of *Halorubrum* archaeorhodopsins—from AR3 to *He*AR, systematically characterized by the authors’ team—is a vivid example of this source supply. With the integration of structure prediction, machine learning and automated functional characterization, a rational design loop “from salt lake resources to customized tools” is taking shape. Its near-term value lies in basic neuroscience, where better silencing tools are still needed; the clinical horizon remains confined to retinal optogenetic therapies based on animal-type opsins and engineered channelrhodopsins, and no archaeorhodopsin—*He*AR included—currently has the neuronal validation that would be required before clinical application could be considered.

## Figures and Tables

**Figure 2:**
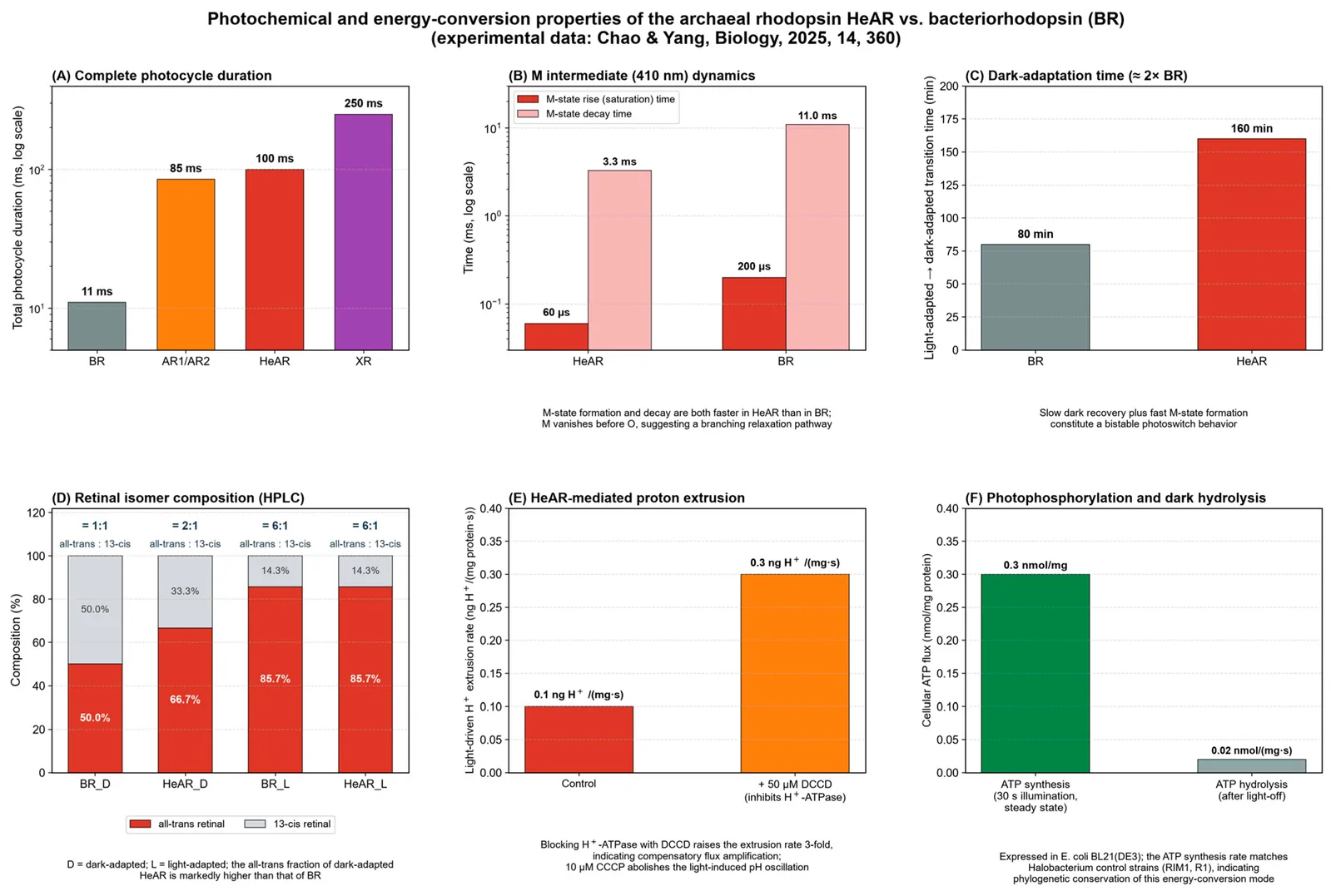
Photocycle kinetics and energy coupling of *He*AR compared with classical ion-pumping rhodopsins (data from the authors’ published work [24]). (**A**) Total photocycle duration (log scale); (**B**) M-state rise and decay times; (**C**) dark-adaptation half-time; (**D**) retinal isomeric composition in the dark- and light-adapted states; (**E**) net proton extrusion rate of the *E. coli* recombinant system and the effect of the ATP synthase inhibitor DCCD; (**F**) light-driven ATP synthesis versus dark ATP hydrolysis. BR, bacteriorhodopsin; AR1/AR2, archaerhodopsin; XR, xanthorhodopsin. Source and copyright note for Figure 2: This figure was drawn by the authors for this manuscript and was not reproduced or adapted from any published figure. All plotted values are taken from the authors’ own open-access article (Ref. [24]: Chao L, Yang Y. Biology 2025, 14, 360; DOI: 10.3390/biology14040360; PMID 40282225), which is published by MDPI under a Creative Commons Attribution 4.0 International License (CC BY 4.0; https://creativecommons.org/licenses/by/4.0/). Reuse of the underlying data is therefore permitted under the terms of that license, and no permission from a third-party copyright holder is required.

**Figure 3:**
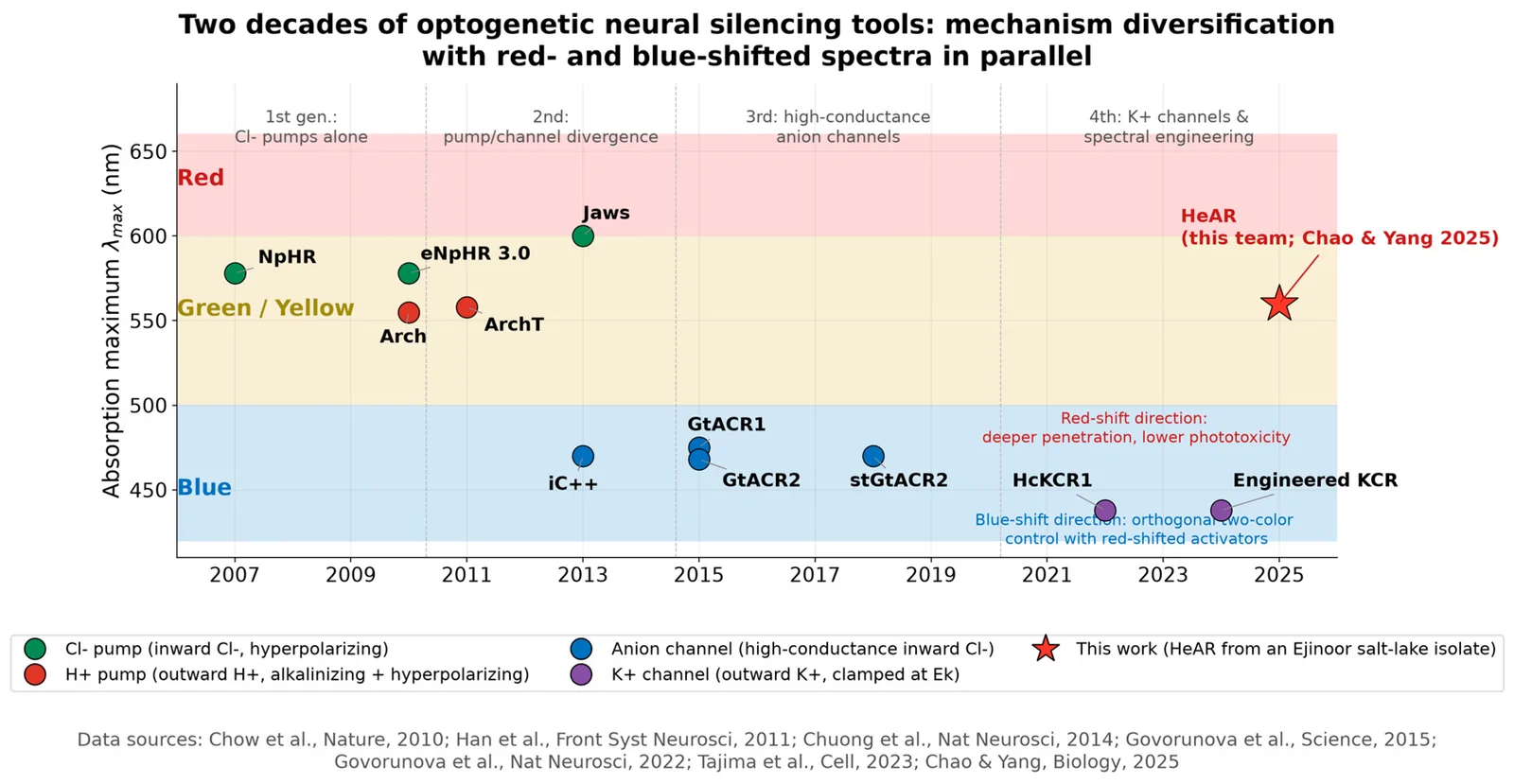
The timeline and mechanistic landscape of optogenetic silencing tools. Top: the evolution of representative silencing tools colored by absorption maximum, including Arch/eNpHR3.0 [20], ArchT [115], Jaws [158], GtACR1/2 [11], HcKCR1 [12], and engineered KCR [48] (the red star marks *He*AR systematically characterized by this group [24]). Bottom: a comparison of the four silencing mechanisms (inward chloride pump, outward proton pump, anion channel, and potassium channel).

**Table 3:** Representative evolution of optogenetic silencing tools.

Tool	Source	Mode of Action	Activation Wavelength/nm	Main Advantages	Main Limitations	Ref.
NpHR	*Natronomonas pharaonis*	Inward Cl^−^ pump	≈578	First genetically encoded optical silencing tool	Poor membrane localization; dependence on intracellular Cl^−^ homeostasis	[8,46,110]
eNpHR3.0	Engineered *Np*HR (with membrane trafficking signal peptide)	Inward Cl^−^ pump	≈585	Markedly improved membrane targeting; red-shifted activation	Risk of Cl^−^ gradient reversal at axon terminals	[20]
Jaws	*Halobacterium salinarum* strain Shark (engineered cruxhalorhodopsin)	Inward Cl^−^ pump	—	Red-shifted cruxhalorhodopsin; red-light photocurrents ≈ 3× those of earlier silencers; enables noninvasive transcranial inhibition of deep neurons in awake mice	Cl^−^-gradient dependence shared with other pump-based silencers	[158]
Arch (AR3)	*Halorubrum sodomense*	Outward H^+^ pump	≈550	Single-gene; effective at low light intensity	Extracellular pH shift; bidirectional reversal effects	[20,97]
ArchT	*Halorubrum* isolate	Outward H^+^ pump	≈550	Markedly higher photosensitivity than Arch	Same as Arch (pH-related side effects)	[113]
iC1C2/iC++	Engineered ChR1/ChR2 chimera	Cl^−^ channel	≈470	Large conductance; fast opening	Residual H^+^ current (eliminated in iC++)	[116,126]
GtACR1/2	*Guillardia theta*	Anion channel	≈470	Natural Cl^−^ selectivity; high unitary conductance	Inactivation depends on continuous illumination	[11]
HcKCR1/2	*Hyphochytrium catenoides*	K^+^ channel	—	P(K)/P(Na) ≈ 23; opens within <1 ms	New tool; long-term characterization needed	[12,48]
eOPN3	Mosquito (insect) brain opsin	Gi/o metabotropic pathway	—	Efficient presynaptic terminal silencing	Slow metabotropic response (seconds)	[114]
*He*AR	*Halorubrum* sp. Ejinoor	Outward H^+^ pump	550 (dark)/560 (light)	550–560 nm window, ≈80–90 nm to the red of excitatory tools (≈470 nm); absorption maximum essentially identical to AR3 (559 nm) and ≈18 nm blue-shifted relative to BR; light-driven ATP synthesis	Relatively slow overall photocycle (100 ms)	[24]

Note: λmax values are approximate; the *He*AR row is based on the authors’ published work [24].

## Data Availability

No new data were created or analyzed in this review. All quantitative values discussed are quoted from the cited publications; the *He*AR measurements are those of the authors’ previously published open-access article (Ref. [24]).

## References

[1] Kojima K., Sudo Y. (2023). Convergent evolution of animal and microbial rhodopsins. RSC Adv..

[2] Ostrovsky M.A., Smitienko O.A., Bochenkova A.V., Feldman T.B. (2023). Similarities and Differences in Photochemistry of Type I and Type II Rhodopsins. Biochemistry.

[3] Oesterhelt D., Stoeckenius W. (1971). Rhodopsin-like protein from the purple membrane of *Halobacterium halobium*. Nat. New Biol..

[4] Hartmann R., Oesterhelt D. (1977). Bacteriorhodopsin-mediated photophosphorylation in *Halobacterium halobium*. Eur. J. Biochem..

[5] Henderson R., Baldwin J.M., Ceska T.A., Zemlin F., Beckmann E., Downing K. (1990). Model for the structure of bacteriorhodopsin based on high-resolution electron cryo-microscopy. J. Mol. Biol..

[6] Luecke H., Schobert B., Richter H.T., Cartailler J.-P., Lanyi J.K. (1999). Structure of bacteriorhodopsin at 1.55 A resolution. J. Mol. Biol..

[7] Schobert B., Lanyi J.K. (1982). Halorhodopsin is a light-driven chloride pump. J. Biol. Chem..

[8] Bamberg E., Tittor J., Oesterhelt D. (1993). Light-driven proton or chloride pumping by halorhodopsin. Proc. Natl. Acad. Sci. USA.

[9] Inoue K., Ono H., Abe-Yoshizumi R., Yoshizawa S., Ito H., Kogure K., Kandori H. (2013). A light-driven sodium ion pump in marine bacteria. Nat. Commun..

[10] Nagel G., Szellas T., Huhn W., Kateriya S., Adeishvili N., Berthold P., Ollig D., Hegemann P., Bamberg E. (2003). Channelrhodopsin-2, a directly light-gated cation-selective membrane channel. Proc. Natl. Acad. Sci. USA.

[11] Govorunova E.G., Sineshchekov O.A., Janz R., Liu X., Spudich J.L. (2015). Natural light-gated anion channels: A family of microbial rhodopsins for advanced optogenetics. Science.

[12] Govorunova E.G., Gou Y., Sineshchekov O.A., Li H., Lu X., Wang Y., Brown L.S., St-Pierre F., Xue M., Spudich J.L. (2022). Kalium channelrhodopsins are natural light-gated potassium channels that mediate optogenetic inhibition. Nat. Neurosci..

[13] Shihoya W., Inoue K., Singh M., Konno M., Hososhima S., Yamashita K., Ikeda K., Higuchi A., Izume T., Okazaki S. (2019). Crystal structure of heliorhodopsin. Nature.

[14] Boyden E.S., Zhang F., Bamberg E., Nagel G., Deisseroth K. (2005). Millisecond-timescale, genetically targeted optical control of neural activity. Nat. Neurosci..

[15] Zhang F., Wang L.P., Boyden E.S., Deisseroth K. (2006). Channelrhodopsin-2 and optical control of excitable cells. Nat. Methods.

[16] Deisseroth K. (2015). Optogenetics: 10 years of microbial opsins in neuroscience. Nat. Neurosci..

[17] Zhang F., Vierock J., Yizhar O., Fenno L.E., Tsunoda S., Kianianmomeni A., Prigge M., Berndt A., Cushman J., Polle J. (2011). The microbial opsin family of optogenetic tools. Cell.

[18] Mattis J., Tye K.M., Ferenczi E.A., Ramakrishnan C., O’Shea D.J., Prakash R., Gunaydin L.A., Hyun M., Fenno L.E., Gradinaru V. (2011). Principles for applying optogenetic tools derived from direct comparative analysis of microbial opsins. Nat. Methods.

[19] Rost B.R., Schneider-Warme F., Schmitz D., Hegemann P. (2017). Optogenetic Tools for Subcellular Applications in Neuroscience. Neuron.

[20] Chow B.Y., Han X., Dobry A.S., Qian X., Chuong A.S., Li M., Henninger M.A., Belfort G.M., Lin Y., Monahan P.E. (2010). High-performance genetically targetable optical neural silencing by light-driven proton pumps. Nature.

[21] Wiegert J.S., Mahn M., Prigge M., Printz Y., Yizhar O. (2017). Silencing Neurons: Tools, Applications, and Experimental Constraints. Neuron.

[22] Rost B.R., Wietek J., Yizhar O., Schmitz D. (2022). Optogenetics at the presynapse. Nat. Neurosci..

[23] Chaoluomeng D.G., Kikukawa T., Ihara K., Iwasa T. (2015). Microbial rhodopsins of *Halorubrum* species isolated from Ejinoor salt lake in Inner Mongolia of China. Photochem. Photobiol. Sci..

[24] Chao L., Yang Y. (2025). Elucidation of expression patterns and functional properties of archaerhodopsin derived from *Halorubrum* sp. Ejinoor. Biology.

[25] Lanyi J.K. (2004). Bacteriorhodopsin. Annu. Rev. Physiol..

[26] Kandori H. (2015). Ion-pumping microbial rhodopsins. Front. Mol. Biosci..

[27] Kovalev K., Polovinkin V., Gushchin I., Alekseev A., Shevchenko V., Borshchevskiy V., Astashkin R., Balandin T., Bratanov D., Vaganova S. (2019). Structure and mechanisms of sodium-pumping KR2 rhodopsin. Sci. Adv..

[28] Engelhard C., Chizhov I., Siebert F., Engelhard M. (2018). Microbial Halorhodopsins: Light-Driven Chloride Pumps. Chem. Rev..

[29] Nakajima Y., Tsukamoto T., Kumagai Y., Ogura Y., Hayashi T., Song J., Kikukawa T., Demura M., Kogure K., Sudo Y. (2018). Presence of a Haloarchaeal Halorhodopsin-Like Cl(-) Pump in Marine Bacteria. Microbes Environ..

[30] Inoue K., Ito S., Kato Y., Nomura Y., Shibata M., Uchihashi T., Tsunoda S.P., Kandori H. (2016). A natural light-driven inward proton pump. Nat. Commun..

[31] Higuchi A., Shihoya W., Konno M., Ikuta T., Kandori H., Inoue K., Nureki O. (2021). Crystal structure of schizorhodopsin reveals mechanism of inward proton pumping. Proc. Natl. Acad. Sci. USA.

[32] Urui T., Hayashi K., Mizuno M., Inoue K., Kandori H., Mizutani Y. (2024). Cis–trans reisomerization preceding reprotonation of the retinal chromophore is common to the schizorhodopsin family: A simple and rational mechanism for inward proton pumping. J. Phys. Chem. B.

[33] Coli A., Gao S., Kaestner L. (2024). Sodium-Selective Channelrhodopsins. Cells.

[34] Kishi K.E., Kim Y.S., Fukuda M., Inoue M., Kusakizako T., Wang P.Y., Ramakrishnan C., Byrne E.F., Thadhani E., Paggi J.M. (2022). Structural basis for channel conduction in the pump-like channelrhodopsin ChRmine. Cell.

[35] Dai G., Geng X. (2018). Photocycle of sensory rhodopsin II from *Halobacterium salinarum* (HsSRII): Mutation of D103 accelerates M decay and changes the decay pathway of a 13-cis O-like species. Photochem. Photobiol..

[36] Matsunami-Nakamura R., Tamogami J., Takeguchi M., Ishikawa J., Kikukawa T., Kamo N., Nara T. (2023). Key determinants for signaling in the sensory rhodopsin II/transducer complex are different between *Halobacterium salinarum* and *Natronomonas pharaonis*. FEBS Lett..

[37] Mukherjee S., Hegemann P., Broser M. (2019). Enzymerhodopsins: Novel photoregulated catalysts for optogenetics. Curr. Opin. Struct. Biol..

[38] Fischer P., Mukherjee S., Schiewer E., Broser M., Bartl F., Hegemann P. (2021). The inner mechanics of rhodopsin guanylyl cyclase during cGMP-formation revealed by real-time FTIR spectroscopy. Elife.

[39] Besaw J.E., Reichenwallner J., De Guzman P., Tucs A., Kuo A., Morizumi T., Tsuda K., Sljoka A., Miller R.J.D., Ernst O.P. (2022). Low pH structure of heliorhodopsin reveals chloride binding site and intramolecular signaling pathway. Sci. Rep..

[40] Tahara S., Singh M., Kuramochi H., Shihoya W., Inoue K., Nureki O., Béjà O., Mizutani Y., Kandori H., Tahara T. (2019). Ultrafast Dynamics of Heliorhodopsins. J. Phys. Chem. B.

[41] Nagata T., Inoue K. (2021). Rhodopsins at a glance. J. Cell Sci..

[42] Gordeliy V., Kovalev K., Bamberg E., Rodriguez-Valera F., Zinovev E., Zabelskii D., Alekseev A., Rosselli R., Gushchin I., Okhrimenko I. (2022). Microbial Rhodopsins. Methods Mol. Biol..

[43] Kandori H. (2021). History and Perspectives of Ion-Transporting Rhodopsins. Adv. Exp. Med. Biol..

[44] Brown L.S. (2022). Light-driven proton transfers and proton transport by microbial rhodopsins—A biophysical perspective. Biochim. Biophys. Acta Biomembr..

[45] Kolbe M., Besir H., Essen L.O., Oesterhelt D. (2000). Structure of the light-driven chloride pump halorhodopsin at 1.8 A resolution. Science.

[46] Kouyama T., Kanada S., Takeguchi Y., Narusawa A., Murakami M., Ihara K. (2010). Crystal structure of the light-driven chloride pump halorhodopsin from *Natronomonas pharaonis*. J. Mol. Biol..

[47] Gushchin I., Shevchenko V., Polovinkin V., Borshchevskiy V., Buslaev P., Bamberg E., Gordeliy V. (2016). Structure of the light-driven sodium pump KR2 and its implications for optogenetics. FEBS J..

[48] Tajima S., Kim Y.S., Fukuda M., Jo Y., Wang P.Y., Paggi J.M., Inoue M., Byrne E.F., Kishi K.E., Nakamura S. (2023). Structural basis for ion selectivity in potassium-selective channelrhodopsins. Cell.

[49] Kataoka M. (2023). Structural studies of bacteriorhodopsin in BC era. Biophys. Physicobiol..

[50] Bratanov D., Kovalev K., Machtens J.P., Astashkin R., Chizhov I., Soloviov D., Volkov D., Polovinkin V., Zabelskii D., Mager T. (2019). Unique structure and function of viral rhodopsins. Nat. Commun..

[51] Kwon S.K., Jun S.H., Kim J.F. (2020). Omega Rhodopsins: A Versatile Class of Microbial Rhodopsins. J. Microbiol. Biotechnol..

[52] Okhrimenko I.S., Kovalev K., Petrovskaya L.E., Ilyinsky N.S., Alekseev A.A., Marin E., Rokitskaya T.I., Antonenko Y.N., Siletsky S.A., Popov P.A. (2023). Mirror proteorhodopsins. Commun. Chem..

[53] Shibata M., Inoue K., Ikeda K., Konno M., Singh M., Kataoka C., Abe-Yoshizumi R., Kandori H., Uchihashi T. (2018). Oligomeric states of microbial rhodopsins determined by high-speed atomic force microscopy and circular dichroic spectroscopy. Sci. Rep..

[54] Ernst O.P., Lodowski D.T., Elstner M., Hegemann P., Brown L.S., Kandori H. (2014). Microbial and animal rhodopsins: Structures, functions, and molecular mechanisms. Chem. Rev..

[55] Gushchin I., Gordeliy V. (2018). Microbial rhodopsins. Subcell. Biochem..

[56] Ihara K., Umemura T., Katagiri I., Kitajima-Ihara T., Sugiyama Y., Kimura Y., Mukohata Y. (1999). Evolution of the archaeal rhodopsins: Evolution rate changes by gene duplication and functional differentiation. J. Mol. Biol..

[57] Sharma A.K., Walsh D.A., Bapteste E., Rodriguez-Valera F., Doolittle W.F., Papke R.T. (2007). Evolution of rhodopsin ion pumps in haloarchaea. BMC Evol. Biol..

[58] Sharma A.K., Spudich J.L., Doolittle W.F. (2006). Microbial rhodopsins: Functional versatility and genetic mobility. Trends Microbiol..

[59] Zabelskii D., Dmitrieva N., Volkov O., Shevchenko V., Kovalev K., Balandin T., Soloviov D., Astashkin R., Zinovev E., Alekseev A. (2021). Structure-based insights into evolution of rhodopsins. Commun. Biol..

[60] Papke R.T., Douady C.J., Doolittle W.F., Rodríguez-Valera F. (2003). Diversity of bacteriorhodopsins in different hypersaline waters from a single Spanish saltern. Environ. Microbiol..

[61] Pinhassi J., DeLong E.F., Béjà O., González J.M., Pedrós-Alió C. (2016). Marine bacterial and archaeal ion-pumping rhodopsins: Genetic diversity, physiology, and ecology. Microbiol. Mol. Biol. Rev..

[62] Frazer S.A., Baghbanzadeh M., Rahnavard A., Crandall K.A., Oakley T.H. (2024). Discovering genotype-phenotype relationships with machine learning and the Visual Physiology Opsin Database (VPOD). Gigascience.

[63] Lanyi J.K., Schobert B. (2007). Structural changes in the L photointermediate of bacteriorhodopsin. J. Mol. Biol..

[64] Soloviov D., Borshchevskiy V., Chizhov I. (2022). Time-Resolved UV-VIS Spectroscopy of Microbial Rhodopsins. Methods Mol. Biol..

[65] Kandori H., Mizutani Y. (2022). FTIR and Raman Spectroscopy of Rhodopsins. Methods Mol. Biol..

[66] Kriebel C.N., Becker-Baldus J., Glaubitz C. (2022). Solid-State NMR Spectroscopy on Microbial Rhodopsins. Methods Mol. Biol..

[67] Maeda A., Iwasa T., Yoshizawa T. (1977). Isomeric composition of retinal chromophore in dark-adapted bacteriorhodopsin. J. Biochem..

[68] Balashov S.P., Imasheva E.S., Govindjee R., Ebrey T.G. (1996). Titration of aspartate-85 in bacteriorhodopsin: What it says about chromophore isomerization and proton release. Biophys. J..

[69] Heberle J., Oesterhelt D., Dencher N.A. (1993). Decoupling of photo- and proton cycle in the. EMBO J..

[70] Subramaniam S., Henderson R. (2000). Molecular mechanism of vectorial proton translocation by bacteriorhodopsin. Nature.

[71] Ge X., Gunner M.R. (2016). Unraveling the mechanism of proton translocation in the extracellular half-channel of bacteriorhodopsin. Proteins.

[72] Weinert T., Skopintsev P., James D., Dworkowski F., Panepucci E., Kekilli D., Furrer A., Brünle S., Mous S., Ozerov D. (2019). Proton uptake mechanism in bacteriorhodopsin captured by serial synchrotron crystallography. Science.

[73] Friedrich D., Brünig F.N., Nieuwkoop A.J., Netz R.R., Hegemann P., Oschkinat H. (2020). Collective exchange processes reveal an active site proton cage in bacteriorhodopsin. Commun. Biol..

[74] Ono J., Okada C., Nakai H. (2022). Hydroxide Ion Mechanism for Long-Range Proton Pumping in the Third Proton Transfer of Bacteriorhodopsin. Chemphyschem.

[75] Yun J.H., Li X., Yue J., Park J.-H., Jin Z., Li C., Hu H., Shi Y., Pandey S., Carbajo S. (2021). Early-stage dynamics of chloride ion-pumping rhodopsin revealed by a femtosecond X-ray laser. Proc. Natl. Acad. Sci. USA.

[76] Siletsky S.A., Mamedov M.D., Lukashev E.P., Balashov S.P., Petrovskaya L.E. (2022). Application of direct electrometry in studies of microbial rhodopsins reconstituted in proteoliposomes. Biophys. Rev..

[77] Yan B., Cline S.W., Doolittle W.F., Spudich J.L. (1992). Transformation of a bop-hop-sop I-sop II- *Halobacterium halobium* mutant to bop+: Effects of bacteriorhodopsin photoactivation on cellular proton fluxes and swimming behavior. Photochem. Photobiol..

[78] Noji T., Ishikita H. (2022). Mechanism of absorption wavelength shift of bacteriorhodopsin during photocycle. J. Phys. Chem. B.

[79] Tsujimura M., Tamura H., Saito K., Ishikita H. (2022). Absorption wavelength along chromophore low-barrier hydrogen bonds. iScience.

[80] Ohashi S., Katayama K., Kojima A., Yang X., Fukuda M., Sacchetta F., Suno R., Sugita Y., Nuemket N., Kim S. (2026). Structural insights into spectral tuning and retinal exchange in cone visual pigments. Science.

[81] Balashov S.P., Imasheva E.S., Boichenko V.A., Antón J., Wang J.M., Lanyi J.K. (2005). Xanthorhodopsin: A proton pump with a light-harvesting carotenoid antenna. Science.

[82] Polívka T., Balashov S.P., Chábera P., Imasheva E.S., Yartsev A., Sundström V., Lanyi J.K. (2009). Femtosecond carotenoid to retinal energy transfer in xanthorhodopsin. Biophys. J..

[83] Imasheva E.S., Balashov S.P., Choi A.R., Jung K.-H., Lanyi J.K. (2009). Reconstitution of Gloeobacter violaceus rhodopsin with a light-harvesting carotenoid antenna. Biochemistry.

[84] Strauss J., Deng L., Gao S., Toseland A., Bachy C., Zhang C., Kirkham A., Hopes A., Utting R., Joest E.F. (2023). Plastid-localized xanthorhodopsin increases diatom biomass and ecosystem productivity in iron-limited surface oceans. Nat. Microbiol..

[85] Petrovskaya L.E., Lukashev E.P., Lyukmanova E.N., Shulepko M.A., Kryukova E.A., Ziganshin R.H., Dolgikh D.A., Maksimov E.G., Rubin A.B., Kirpichnikov M.P. (2023). Expression of Xanthorhodopsin in *Escherichia coli*. Protein J..

[86] Petrovskaya L.E., Bolshakov V.A., Lukashev E.P., Kryukova E.A., Maksimov E.G., Rubin A.B., Dolgikh D.A., Balashov S.P., Kirpichnikov M.P. (2025). Engineering of thermal stability in the recombinant xanthorhodopsin from Salinibacter ruber. Biochim. Biophys. Acta Bioenerg..

[87] Boichenko V.A., Wang J.M., Antón J., Lanyi J.K., Balashov S.P. (2006). Functions of carotenoids in xanthorhodopsin and archaerhodopsin, from action spectra of photoinhibition of cell respiration. Biochim. Biophys. Acta.

[88] Fujimoto K.J., Tsuzuki Y.A., Konno M., Nagata T., Murakami R., Inoue K., Yanai T. (2025). Protonation-Enhanced Energy Transfer in Xanthorhodopsin Kin4B8. J. Phys. Chem. Lett..

[89] Ghosh M., Misra R., Bhattacharya S., Majhi K., Jung K.-H., Sheves M. (2023). Retinal-Carotenoid Interactions in a Sodium-Ion-Pumping Rhodopsin: Implications on Oligomerization and Thermal Stability. J. Phys. Chem. B.

[90] Kopejtka K., Tomasch J., Zeng Y., Selyanin V., Dachev M., Piwosz K., Tichý M., Bína D., Gardian Z., Bunk B. (2020). Simultaneous Presence of Bacteriochlorophyll and Xanthorhodopsin Genes in a Freshwater Bacterium. mSystems.

[91] Straková D., Sánchez-Porro C., de la Haba R.R., Ventosa A. (2025). Strategies of Environmental Adaptation in the Haloarchaeal Genera Haloarcula and Natrinema. Microorganisms.

[92] Soto L., DasSarma P., Anton B.P., Vincze T., Verma I., Eralp B., Powers D.W., Dozier B.L., Roberts R.J., DasSarma S. (2024). Genome sequence of an extremely halophilic archaeon isolated from Permian Period halite, Salado Formation in New Mexico, USA: Halobacterium sp. strain NMX12-1. Microbiol. Resour. Announc..

[93] Peck R.F., Pleşa A.M., Graham S.M., Angelini D.R., Shaw E.L. (2017). Opsin-Mediated Inhibition of Bacterioruberin Synthesis in Halophilic Archaea. J. Bacteriol..

[94] Peck R.F., Graham S.M., Gregory A.M. (2019). Species Widely Distributed in Halophilic Archaea Exhibit Opsin-Mediated Inhibition of Bacterioruberin Biosynthesis. J. Bacteriol..

[95] Sugiyama Y., Maeda M., Futai M., Mukohata Y. (1989). Isolation of a gene that encodes a new retinal protein, archaerhodopsin, from Halobacterium sp. aus-1. J. Biol. Chem..

[96] Uegaki K., Sugiyama Y., Mukohata Y. (1991). Archaerhodopsin-2, from Halobacterium sp. aus-2 further reveals essential amino acid residues for light-driven proton pumps. Arch. Biochem. Biophys..

[97] Lukashev E.P., Govindjee R., Kono M., Ebrey T.G., Sugiyama Y., Mukohata Y. (1994). PH dependence of the absorption spectra and photochemical transformations of the archaerhodopsins. Photochem. Photobiol..

[98] Feng J., Liu H.C., Chu J.F., Zhou P.J., Tang J.A., Liu S.J. (2006). Genetic cloning and functional expression in *Escherichia coli* of an archaerhodopsin gene from *Halorubrum xinjiangense*. Extremophiles.

[99] Bada Juarez J.F., Judge P.J., Adam S., Axford D., Vinals J., Birch J., Kwan T.O.C., Hoi K.K., Yen H.-Y., Vial A. (2021). Structures of the archaerhodopsin-3 transporter reveal that disordering of internal water networks underpins receptor sensitization. Nat. Commun..

[100] Geng X., Dai G., Chao L., Wen D., Kikukawa T., Iwasa T. (2019). Two consecutive polar amino acids at the end of helix E are important for fast turnover of the archaerhodopsin photocycle. Photochem. Photobiol..

[101] Herasymenko K., Walisinghe D., Konno M., Barneschi L., de Waele I., Sliwa M., Inoue K., Olivucci M., Haacke S. (2025). Archaerhodopsin 3 is an ideal template for the engineering of highly fluorescent optogenetic reporters. Chem. Sci..

[102] Herasymenko K., Walisinghe D., Konno M., Ledentu V., Huix-Rotllant M., Yang X., Ferré N., Inoue K., Olivucci M., Haacke S. (2026). Effects of counter ion protonation on the ultrafast excited-state dynamics in archaerhodopsin-3. Biophys. J..

[103] Maclaurin D., Venkatachalam V., Lee H., Cohen A.E. (2013). Mechanism of voltage-sensitive fluorescence in a microbial rhodopsin. Proc. Natl. Acad. Sci. USA.

[104] Ming M., Lu M., Balashov S.P., Ebrey T.G., Li Q., Ding J. (2006). PH dependence of light-driven proton pumping by an archaerhodopsin from Tibet: Comparison with bacteriorhodopsin. Biophys. J..

[105] Wang Y., Wu J., Ming M., Zhao Y., Ding J. (2012). Effects of Triton X-100 on proton transfer and in the photocycle of archaerhodopsin 4. Biosci. Biotechnol. Biochem..

[106] Cao Z., Ding X., Peng B., Zhao Y., Ding J., Watts A., Zhao X. (2015). Novel expression and characterization of a light driven proton pump archaerhodopsin 4 in a *Halobacterium salinarum* strain. Biochim. Biophys. Acta.

[107] Ganapathy S., Kratz S., Chen Q., Hellingwerf K.J., de Groot H.J., Rothschild K.J., de Grip W.J. (2019). Redshifted and Near-infrared Active Analog Pigments Based upon Archaerhodopsin-3. Photochem. Photobiol..

[108] Gong Y., Li J.Z., Schnitzer M.J. (2013). Enhanced Archaerhodopsin Fluorescent Protein Voltage Indicators. PLoS ONE.

[109] Chien M.P., Brinks D., Testa-Silva G., Tian H., Brooks F.P., Adam Y., Bloxham W., Gmeiner B., Kheifets S., Cohen A.E. (2021). Photoactivated voltage imaging in tissue with an archaerhodopsin-derived reporter. Sci. Adv..

[110] El-Gaby M., Zhang Y., Wolf K., Schwiening C.J., Paulsen O., Shipton O.A. (2016). Archaerhodopsin Selectively and Reversibly Silences Synaptic Transmission through Altered pH. Cell Rep..

[111] Nakao S., Kojima K., Sudo Y. (2022). Phototriggered Apoptotic Cell Death (PTA) Using the Light-Driven Outward Proton Pump Rhodopsin Archaerhodopsin-3. J. Am. Chem. Soc..

[112] Sun C., Ding X., Cui H., Yang Y., Chen S., Watts A., Zhao X. (2018). In situ study of the function of bacterioruberin in the dual-chromophore photoreceptor archaerhodopsin-4. Angew. Chem. Int. Ed..

[113] Gradinaru V., Thompson K.R., Zhang F., Mogri M., Kay K., Schneider M.B., Deisseroth K. (2007). Targeting and readout strategies for fast optical neural control in vitro and in vivo. J. Neurosci..

[114] Feroz H., Ferlez B., Lefoulon C., Ren T., Baker C.S., Gajewski J.P., Lugar D.J., Gaudana S.B., Butler P.J., Hühn J. (2018). Light-Driven Chloride Transport Kinetics of Halorhodopsin. Biophys. J..

[115] Han X., Chow B.Y., Zhou H., Klapoetke N.C., Chuong A., Rajimehr R., Yang A., Baratta M.V., Winkle J., Desimone R. (2011). A high-light sensitivity optical neural silencer: Development and application to optogenetic control of non-human primate cortex. Front. Syst. Neurosci..

[116] Lehtinen K., Nokia M.S., Takala H. (2021). Red Light Optogenetics in Neuroscience. Front. Cell. Neurosci..

[117] Berndt A., Lee S.Y., Ramakrishnan C., Deisseroth K. (2014). Structure-guided transformation of channelrhodopsin into a light-activated chloride channel. Science.

[118] Klapoetke N.C., Murata Y., Kim S.S., Pulver S.R., Birdsey-Benson A., Cho Y.K., Morimoto T.K., Chuong A.S., Carpenter E.J., Tian Z. (2014). Independent optical excitation of distinct neural populations. Nat. Methods.

[119] Hegemann P., Möglich A. (2011). Channelrhodopsin engineering and exploration of new optogenetic tools. Nat. Methods.

[120] Rappleye M., Berndt A. (2019). Structural basis for ion selectivity and engineering in channelrhodopsins. Curr. Opin. Struct. Biol..

[121] Kato H.E. (2021). Structure-Function Relationship of Channelrhodopsins. Adv. Exp. Med. Biol..

[122] Tsujimura M., Kojima K., Kawanishi S., Sudo Y., Ishikita H. (2021). Proton transfer pathway in anion channelrhodopsin-1. Elife.

[123] Govorunova E.G., Sineshchekov O.A., Spudich J.L. (2023). Potassium-selective channelrhodopsins. Biophys. Physicobiol.

[124] Bernal Sierra Y.A., Rost B.R., Pofahl M., Fernandes A.M., Kopton R.A., Moser S., Holtkamp D., Masala N., Beed P., Tukker J.J. (2018). Potassium channel-based optogenetic silencing. Nat. Commun..

[125] Wietek J., Nozownik A., Pulin M., Saraf-Sinik I., Matosevich N., Gowrishankar R., Gat A., Malan D., Brown B.J., Dine J. (2024). A bistable inhibitory optoGPCR for multiplexed optogenetic control of neural circuits. Nat. Methods.

[126] Mahn M., Saraf-Sinik I., Patil P., Pulin M., Bitton E., Karalis N., Bruentgens F., Palgi S., Gat A., Dine J. (2021). Efficient optogenetic silencing of neurotransmitter release with a mosquito rhodopsin. Neuron.

[127] Spreen A., Sadanandan N.P., Schneider M.W., Kuehn E., Leemisa A.N., De Zio R., Meyer N., Busse W., Erlinghagen B., Adenauer L. (2025). Optogenetic silencing by combining a rhodopsin cyclase with an engineered cGMP-gated potassium channel. Sci. Adv..

[128] Vierock J., Rodriguez-Rozada S., Dieter A., Pieper F., Sims R., Tenedini F., Bergs A.C.F., Bendifallah I., Zhou F., Zeitzschel N. (2021). BiPOLES is an optogenetic tool developed for bidirectional dual-color control of neurons. Nat. Commun..

[129] Shimoda Y., Beppu K., Ikoma Y., Morizawa Y.M., Zuguchi S., Hino U., Yano R., Sugiura Y., Moritoh S., Fukazawa Y. (2022). Optogenetic stimulus-triggered acquisition of seizure resistance. Neurobiol. Dis..

[130] Walker M.C., Kullmann D.M. (2020). Optogenetic and chemogenetic therapies for epilepsy. Neuropharmacology.

[131] Kravchenko J.A., Goldberg E.M., Mattis J. (2023). Optogenetic and chemogenetic manipulation of seizure threshold in mice. STAR Protoc..

[132] Bentley J.N., Chestek C., Stacey W.C., Patil P.G. (2013). Optogenetics in epilepsy. Neurosurg. Focus.

[133] Amtul Z., Aziz A.A. (2017). Microbial proteins as novel industrial biotechnology hosts to treat epilepsy. Mol. Neurobiol..

[134] Li G., Zhang C., Li Y., Yang J., Wu J., Shao Y., Ma K., Zhang X., Zhu S., Du J. (2025). Optogenetic vagal nerve stimulation attenuates heart failure by limiting the generation of monocyte-derived inflammatory CCRL2^+^ macrophages. Immunity.

[135] Han Y., Wei X., Chen G., Shao E., Zhou Y., Li Y., Xiao Z., Shi X., Zheng H., Huang S. (2024). Optogenetic Stimulation of the Cardiac Vagus Nerve to Promote Heart Regenerative Repair after Myocardial Infarction. Int. J. Biol. Sci..

[136] Boyle P.M., Karathanos T.V., Trayanova N.A. (2018). Cardiac Optogenetics: 2018. JACC Clin. Electrophysiol..

[137] Alekseev A., Hunniford V., Zerche M., Jeschke M., El May F., Vavakou A., Siegenthaler D., Hüser M.A., Kiehn S.M., Garrido-Charles A. (2026). Efficient and sustained optogenetic control of sensory and cardiac systems. Nat. BioMed. Eng..

[138] Abilez O.J. (2012). Cardiac optogenetics. Annu. Int. Conf. IEEE Eng. Med. Biol. Soc..

[139] Guo W., Hight A.E., Chen J.X., Klapoetke N.C., Hancock K.E., Shinn-Cunningham B.G., Boyden E.S., Lee D.J., Polley D.B. (2015). Hearing the light: Neural and perceptual encoding of optogenetic stimulation in the central auditory pathway. Sci. Rep..

[140] Huet A.T., Dombrowski T., Rankovic V., Thirumalai A., Moser T. (2021). Developing Fast, Red-Light Optogenetic Stimulation of Spiral Ganglion Neurons for Future Optical Cochlear Implants. Front. Mol. Neurosci..

[141] Iyer S.M., Vesuna S., Ramakrishnan C., Huynh K., Young S., Berndt A., Lee S.Y., Gorini C.J., Deisseroth K., Delp S.L. (2016). Optogenetic and chemogenetic strategies for sustained inhibition of pain. Sci. Rep..

[142] Yi M.H., Liu Y., Liu Y.U., Lee J., Hanumaihgari P., Parusel S., Bosco D.B., Wang L., Zheng J., Shi W. (2025). Optogenetic activation of cortical microglia promotes neuronal activity and pain hypersensitivity. Cell Rep..

[143] De Luca R., Nardone S., Grace K.P., Venner A., Cristofolini M., Bandaru S.S., Sohn L.T., Kong D., Mochizuki T., Viberti B. (2022). Orexin neurons inhibit sleep to promote arousal. Nat. Commun..

[144] Zhao Q., Maci M., Miller M.R., Zhou H., Zhang F., Algamal M., Lee Y.F., Hou S.S., Perle S.J., Le H. (2023). Sleep restoration by optogenetic targeting of GABAergic neurons reprograms microglia and ameliorates pathological phenotypes in an Alzheimer’s disease model. Mol. Neurodegener..

[145] Qin H., Fu L., Jian T., Jin W., Liang M., Li J., Chen Q., Yang X., Du H., Liao X. (2022). REM sleep-active hypothalamic neurons may contribute to hippocampal social-memory consolidation. Neuron.

[146] Luo Y.J., Li Y.D., Wang L., Yang S.-R., Yuan X.-S., Wang J., Cherasse Y., Lazarus M., Chen J.-F., Qu W.-M. (2018). Nucleus accumbens controls wakefulness by a subpopulation of neurons expressing dopamine D(1) receptors. Nat. Commun..

[147] Duebel J., Marazova K., Sahel J.A. (2015). Optogenetics. Curr. Opin. Ophthalmol..

[148] Pan Z.H., Lu Q., Bi A., Dizhoor A.M., Abrams G.W. (2015). Optogenetic Approaches to Restoring Vision. Annu. Rev. Vis. Sci..

[149] Henriksen B.S., Marc R.E., Bernstein P.S. (2014). Optogenetics for retinal disorders. J. Ophthalmic Vis. Res..

[150] Cehajic-Kapetanovic J., Eleftheriou C., Allen A.E., Milosavljevic N., Pienaar A., Bedford R., Davis K.E., Bishop P.N., Lucas R.J. (2015). Restoration of Vision with Ectopic Expression of Human Rod Opsin. Curr. Biol..

[151] Mohanty S.K., Mahapatra S., Batabyal S., Carlson M., Kanungo G., Ayyagari A., Tchedre K., Franco J.A., Singer M., Barone S.B. (2025). A synthetic opsin restores vision in patients with severe retinal degeneration. Mol. Ther..

[152] Lam B.L., Zak V., Gonzalez V.H., Gregori N.Z., Chavala S.H., Batabyal S., Carlson M., Kim S., Ayyagari A., Chang J. (2025). Safety and efficacy of MCO-010 optogenetic therapy in patients with Stargardt disease in USA (STARLIGHT): An open-label multi-center Ph2 trial. EClinicalMedicine.

[153] Burnight E.R., Wiley L.A., Mullin N.K., Adur M.K., Lang M.J., Cranston C.M., Jiao C., Russell S.R., Sohn E.H., Han I.C. (2023). CRISPRi-Mediated Treatment of Dominant Rhodopsin-Associated Retinitis Pigmentosa. Cris. J..

[154] Busskamp V., Roska B., Sahel J.A. (2024). Optogenetic Vision Restoration. Cold Spring Harb. Perspect. Med..

[155] Scalabrino M.L., Field G.D. (2023). Neuroscience: Visual restoration with optogenetics. Curr. Biol..

[156] Hughes S., Rodgers J., Lindner M., Peirson S.N., Lucas R.J., Hankins M.W. (2025). Optogenetic vision restoration in the face of secondary and tertiary remodeling in the rd1 mouse retina. Mol. Ther..

[157] Lu Q., Pan Z.H. (2021). Optogenetic Strategies for Vision Restoration. Adv. Exp. Med. Biol..

[158] Chuong A.S., Miri M.L., Busskamp V., Matthews G.A.C., Acker L.C., Sørensen A.T., Young A., Klapoetke N.C., A Henninger M., Kodandaramaiah S.B. (2014). Noninvasive optical inhibition with a red-shifted microbial rhodopsin. Nat. Neurosci..

[159] Jumper J., Evans R., Pritzel A., Green T., Figurnov M., Ronneberger O., Tunyasuvunakool K., Bates R., Žídek A., Potapenko A. (2021). Highly accurate protein structure prediction with AlphaFold. Nature.

[160] Pettersen E.F., Goddard T.D., Huang C.C., Meng E.C., Couch G.S., Croll T.I., Morris J.H., Ferrin T.E. (2021). UCSF ChimeraX: Structure visualization for researchers, educators, and developers. Protein Sci..

[161] Inoue K., Del Carmen Marín M., Tomida S., Nakamura R., Nakajima Y., Olivucci M., Kandori H. (2019). Red-shifting mutation of light-driven sodium-pump rhodopsin. Nat. Commun..

[162] Cho S.G., Shim J.G., Choun K., Meas S., Kang K.-W., Kim J.-H., Cho H.-S., Jung K.-H. (2021). Discovery of a new light-driven Li^+^/Na^+^-pumping rhodopsin with DTG motif. J. Photochem. Photobiol. B.

[163] Murabe K., Tsukamoto T., Aizawa T., Demura M., Kikukawa T. (2020). Direct Detection of the Substrate Uptake and Release Reactions of the Light-Driven Sodium-Pump Rhodopsin. J. Am. Chem. Soc..

[164] Kaur J., Kriebel C.N., Eberhardt P., Jakdetchai O., Leeder A.J., Weber I., Brown L.J., Brown R.C., Becker-Baldus J., Bamann C. (2019). Solid-state NMR analysis of the sodium pump Krokinobacter rhodopsin 2 and its H30A mutant. J. Struct. Biol..

[165] Tsunoda S.P., Prigge M., Abe-Yoshizumi R., Inoue K., Kozaki Y., Ishizuka T., Yawo H., Yizhar O., Kandori H. (2017). Functional characterization of sodium-pumping rhodopsins with different pumping properties. PLoS ONE.

[166] Henrich E., Sörmann J., Eberhardt P., Peetz O., Mezhyrova J., Morgner N., Fendler K., Dötsch V., Wachtveitl J., Bernhard F. (2017). From Gene to Function: Cell-Free Electrophysiological and Optical Analysis of Ion Pumps in Nanodiscs. Biophys. J..

[167] Tucker K., Sridharan S., Adesnik H., Brohawn S.G. (2022). Cryo-EM structures of the channelrhodopsin ChRmine in lipid nanodiscs. Nat. Commun..

[168] Tanaka T., Hososhima S., Yamashita Y., Sugimoto T., Nakamura T., Shigemura S., Iida W., Sano F.K., Oda K., Uchihashi T. (2024). The high-light-sensitivity mechanism and optogenetic properties of the bacteriorhodopsin-like channelrhodopsin GtCCR4. Mol. Cell.

[169] Abraham M.J., Murtola T., Schulz R., Páll S., Smith J.C., Hess B., Lindahl E. (2015). GROMACS: High performance molecular simulations through multi-level parallelism from laptops to supercomputers. SoftwareX.

[170] Hess B., Kutzner C., van der Spoel D., Lindahl E. (2008). GROMACS 4: Algorithms for highly efficient, load-balanced, and scalable molecular simulation. J. Chem. Theory Comput..

[171] Trott O., Olson A.J. (2010). AutoDock Vina: Improving the speed and accuracy of docking with a new scoring function, efficient optimization, and multithreading. J. Comput. Chem..

[172] Grünbein M.L., Kovacs G.N., Kloos M., Gorel A., Doak R.B., Shoeman R.L., Barends T.R.M., Schlichting I. (2022). Crystallographic Studies of Rhodopsins: Structure and Dynamics. Methods Mol. Biol..

[173] Picarazzi F., Manetti F., Marigo V., Marigo V., Mori M. (2022). Conformational insights into the C-terminal mutations of human rhodopsin in retinitispigmentosa. J. Mol. Graph. Model..

[174] Kato H.E., Kamiya M., Sugo S., Ito J., Taniguchi R., Orito A., Hirata K., Inutsuka A., Yamanaka A., Maturana A.D. (2015). Atomistic design of microbial opsin-based blue-shifted optogenetics tools. Nat. Commun..

[175] Romero P.A., Arnold F.H. (2009). Exploring protein fitness landscapes by directed evolution. Nat. Rev. Mol. Cell Biol..

[176] McIsaac R.S., Engqvist M.K., Wannier T., Rosenthal A.Z., Herwig L., Flytzanis N.C., Imasheva E.S., Lanyi J.K., Balashov S.P., Gradinaru V. (2014). Directed evolution of a far-red fluorescent rhodopsin. Proc. Natl. Acad. Sci. USA.

[177] Herwig L., Rice A.J., Bedbrook C.N., Zhang R.K., Lignell A., Cahn J.K., Renata H., Dodani S.C., Cho I., Cai L. (2017). Directed Evolution of a Bright Near-Infrared Fluorescent Rhodopsin Using a Synthetic Chromophore. Cell Chem. Biol..

[178] Engqvist M.K., McIsaac R.S., Dollinger P., Flytzanis N.C., Abrams M., Schor S., Arnold F.H. (2015). Directed evolution of Gloeobacter violaceus rhodopsin spectral properties. J. Mol. Biol..

[179] Nikolaev A., Orlov Y., Tsybrov F., Kuznetsova E., Shishkin P., Kuzmin A., Mikhailov A., Nikolaeva Y.S., Anuchina A., Chizhov I. (2025). Engineering of soluble bacteriorhodopsin. Chem. Sci..

[180] Sasaki J., Brown L.S., Chon Y.S., Kandori H., Maeda A., Needleman R., Lanyi J.K. (1995). Conversion of bacteriorhodopsin into a chloride ion pump. Science.

[181] Kawanabe A., Furutani Y., Jung K.H., Kandori H. (2009). Engineering an inward proton transport from a bacterial sensor rhodopsin. J. Am. Chem. Soc..

[182] Bedbrook C.N., Yang K.K., Robinson J.E., Mackey E.D., Gradinaru V., Arnold F.H. (2019). Machine learning-guided channelrhodopsin engineering enables minimally invasive optogenetics. Nat. Methods.

[183] Karasuyama M., Inoue K., Nakamura R., Kandori H., Takeuchi I. (2018). Understanding Colour Tuning Rules and Predicting Absorption Wavelengths of Microbial Rhodopsins by Data-Driven Machine-Learning Approach. Sci. Rep..

[184] Sela M., Church J.R., Schapiro I., Schneidman-Duhovny D. (2024). RhoMax: Computational Prediction of Rhodopsin Absorption Maxima Using Geometric Deep Learning. J. Chem. Inf. Model..

[185] Frazer S.A., Oakley T.H. (2026). Accessible and robust machine learning approaches to improve the opsin genotype-phenotype map. Mol. Biol. Evol..

[186] Selvaraj M.K., Thakur A., Kumar M., Pinnaka A.K., Suri C.R., Siddhardha B., Elumalai S.P. (2023). Ion-pumping microbial rhodopsin protein classification by machine learning approach. BMC Bioinform..

[187] Singh D., Chuang C., Brumer P. (2024). Machine Learning Optimization of Non-Kasha Behavior and of Transient Dynamics in Model Retinal Isomerization. J. Phys. Chem. Lett..

[188] Johnson T.J., Gakhar S., Risbud S.H., Longo M.L., Johnson K.E. (2018). Development and characterization of titanium dioxide gel with encapsulated bacteriorhodopsin for hydrogen production. Langmuir.

[189] Lv Y., Liang D., Lu S., Aurbach D., Xiang Y. (2021). Unidirectional electron injection and accelerated proton transport in bacteriorhodopsin based Bio-p-n junctions. Biosens. Bioelectron..

[190] Seyedkarimi M.S., Aramvash A., Ramezani R. (2015). High production of bacteriorhodopsin from wild type *Halobacterium salinarum*. Extremophiles.

[191] Kawatake S., Umegawa Y., Matsuoka S., Murata M., Sonoyama M. (2016). Evaluation of diacyl phospholipids as boundary lipids for bacteriorhodopsin from structural and functional aspects. Biochim. Biophys. Acta.

[192] Chen S., Ding X., Sun C., Wang F., He X., Watts A., Zhao X. (2022). Archaeal lipids regulating the trimeric structure dynamics of bacteriorhodopsin for efficient proton release and uptake. Int. J. Mol. Sci..

[193] Vlasova A.D., Bukhalovich S.M., Bagaeva D.F., Polyakova A.P., Ilyinsky N.S., Nesterov S.V., Tsybrov F.M., Bogorodskiy A.O., Zinovev E.V., Mikhailov A.E. (2024). Intracellular microbial rhodopsin-based optogenetics to control metabolism and cell signaling. Chem. Soc. Rev..

[194] Zhang H., Fang H., Liu D., Zhang Y., Adu-Amankwaah J., Yuan J., Tan R., Zhu J. (2022). Applications and challenges of rhodopsin-based optogenetics in biomedicine. Front. Neurosci..

[195] Repina N.A., Rosenbloom A., Mukherjee A., Schaffer D.V., Kane R.S. (2017). At light speed: Advances in optogenetic systems for regulating cell signaling and behavior. Annu. Rev. Chem. Biomol. Eng..

[196] Kawano F., Suzuki H., Furuya A., Sato M. (2015). Engineered pairs of distinct photoswitches for optogenetic control of cellular proteins. Nat. Commun..

[197] Jeong J., Jung J., Jung D. (2021). An implantable optogenetic stimulator wirelessly powered by flexible photovoltaics with near-infrared (NIR) light. Biosens. Bioelectron..

[198] Passmore J.B., Rates A., Schröder J., van Laarhoven M.T.P., Hellebrekers V.J.W., van Hoef H.G., Geurts A.J.M., van Straaten W., Nijenhuis W., Berger F. (2025). Closed-loop optogenetic control of cell biology enables outcome-driven microscopy. Nat. Commun..

[199] Ullrich S., Gueta R., Nagel G. (2013). Degradation of channelopsin-2 in the absence of retinal and degradation resistance in certain mutants. Biol. Chem..

[200] Cubizolle A., Cia D., Moine E., Jacquemot N., Guillou L., Rosell M., Angebault-Prouteau C., Lenaers G., Meunier I., Vercauteren J. (2020). Isopropyl-phloroglucinol-DHA protects outer retinal cells against lethal dose of all-trans-retinal. J. Cell. Mol. Med..

[201] Pasqualetto G., Pileggi E., Schepelmann M., Varricchio C., Rozanowska M., Brancale A., Bassetto M. (2021). Ligand-based rational design, synthesis and evaluation of novel potential chemical chaperones for opsin. Eur. J. Med. Chem..

[202] DuBois D.W., Murchison D.A., Mahnke A.H., Bang E., Winzer-Serhan U., Griffith W.H., Souza K.A. (2023). Maintenance of optogenetic channel rhodopsin (ChR2) function in aging mice: Implications for pharmacological studies of inhibitory synaptic transmission, quantal content, and calcium homeostasis. Neuropharmacology.

[203] Kuwasaki Y., Suzuki K., Yu G., Yamamoto S., Otabe T., Kakihara Y., Nishiwaki M., Miyake K., Fushimi K., Bekdash R. (2022). A red light-responsive photoswitch for deep tissue optogenetics. Nat. Biotechnol..

[204] Zhang L., Liu Y., Luo G., Chen C., Dou C., Du J., Xie H., Guan Y., Yang J., Ding Z. (2024). Upconversion-Mediated Optogenetics for the Treatment of Surgery-Induced Postoperative Neurocognitive Dysfunction. ACS Nano.

[205] Kaberniuk A.A., Baloban M., Monakhov M.V., Shcherbakova D.M., Verkhusha V.V. (2021). Single-component near-infrared optogenetic systems for gene transcription regulation. Nat. Commun..

[206] Karasev M.M., Verkhusha V.V., Shcherbakova D.M. (2023). Near-Infrared Optogenetic Module for Conditional Protein Splicing. J. Mol. Biol..

[207] Berglund K., Stern M.A., Gross R.E. (2021). Bioluminescence-Optogenetics. Adv. Exp. Med. Biol..

[208] Grimm C., Sims R.R., Tanese D., Lafirdeen A.S.M., Bendifallah I., Chan C.Y., Faini G., Putti E., Del Bene F., Papagiakoumou E. (2026). Two-photon voltage imaging with rhodopsin-based sensors. Neuron.

[209] Lakunina A.A., Socha K.Z., Ladd A.E., Bowen A.J., Chen S., Colonell J., Doshi A., Karsh B., Krumin M., Kulik P. (2026). Neuropixels Opto: Combining high-resolution electrophysiology and optogenetics. Nat. Methods.

[210] Ding J., Lu J., Zhang Q., Xu Y., Song B., Wu Y., Shi H., Chu B., Wang H., He Y. (2023). Camouflage Nanoparticles Enable in Situ Bioluminescence-Driven Optogenetic Therapy of Retinoblastoma. ACS Nano.

[211] Vogt M., Schulz B., Wagdi A., Lebert J., van Belle G.J., Christoph J., Bruegmann T., Patejdl R. (2021). Direct optogenetic stimulation of smooth muscle cells to control gastric contractility. Theranostics.

[212] Soor N.S., Quicke P., Howe C.L., Pang K.T., Neil M.A., Schultz S.R., Foust A.J. (2019). All-optical crosstalk-free manipulation and readout of Chronos-expressing neurons. J. Phys. D Appl. Phys..

[213] Abdelfattah A.S., Zheng J., Singh A., Huang Y.-C., Reep D., Tsegaye G., Tsang A., Arthur B.J., Rehorova M., Olson C.V. (2023). Sensitivity optimization of a rhodopsin-based fluorescent voltage indicator. Neuron.

[214] Han Y., Yang J., Li Y., Chen Y., Ren H., Ding R., Qian W., Ren K., Xie B., Deng M. (2023). Bright and sensitive red voltage indicators for imaging action potentials in brain slices and pancreatic islets. Sci. Adv..

[215] Yiangou L., Blanch-Asensio A., de Korte T., Miller D.C., van Meer B.J., Mol M.P.H., Brink L.v.D., Brandão K.O., Mummery C.L., Davis R.P. (2022). Optogenetic Reporters Delivered as mRNA Facilitate Repeatable Action Potential and Calcium Handling Assessment in Human iPSC-Derived Cardiomyocytes. Stem Cells.

[216] Bansal A., Shikha S., Zhang Y. (2023). Towards translational optogenetics. Nat. BioMed. Eng..

[217] Tang F., Yan F., Zhong Y., Li J., Gong H., Li X. (2024). Optogenetic Brain-Computer Interfaces. Bioengineering.

[218] Christie J.M., Zurbriggen M.D. (2021). Optogenetics in plants. New Phytol..

[219] Kohsaka H., Nose A. (2021). Optogenetics in Drosophila. Adv. Exp. Med. Biol..

[220] Rodriguez-Valera F., Pushkarev A., Rosselli R., Béjà O. (2022). Searching Metagenomes for New Rhodopsins. Methods Mol. Biol..

[221] Cifuentes-Anticevic J., Alcamán-Arias M.E., Alarcón-Schumacher T., Tamayo-Leiva J., Pedrós-Alió C., Farías L., Díez B. (2021). Proteorhodopsin Phototrophy in Antarctic Coastal Waters. mSphere.

[222] Kovalev K., Stetsenko A., Trunk F., Marin E., Haro-Moreno J.M., Lamm G.H., Alekseev A., Rodriguez-Valera F., Schneider T.R., Wachtveitl J. (2025). Structural basis for no retinal binding in flotillin-associated rhodopsins. Structure.

[223] Wang Y., Xu J., Cui D., Kong L., Chen S., Xie W., Zhang C. (2021). Classification and Identification of Archaea Using Single-Cell Raman Ejection and Artificial Intelligence: Implications for Investigating Uncultivated Microorganisms. Anal. Chem..

